# Design Maps for the Hyperthermic Treatment of Tumors with Superparamagnetic Nanoparticles

**DOI:** 10.1371/journal.pone.0057332

**Published:** 2013-02-25

**Authors:** Antonio Cervadoro, Chiara Giverso, Rohit Pande, Subhasis Sarangi, Luigi Preziosi, Jarek Wosik, Audrius Brazdeikis, Paolo Decuzzi

**Affiliations:** 1 Department of Translational Imaging, The Methodist Hospital Research Institute, Houston, Texas, United States of America; 2 Department of Mechanics, Politecnico di Torino, Turin, Italy; 3 Department of Mathematical Sciences, Politecnico di Torino, Turin, Italy; 4 Department of Electrical and Computer Engineering, University of Houston, Houston, Texas, United States of America; 5 Texas Superconductivity Center, Houston, Texas, United States of America; 6 Department of Physics, University of Houston, Houston, Texas, United States of America; 7 Department of Experimental and Clinical Medicine, University of “Magna Graecia”, Catanzaro, Italy; Case Western Reserve University, United States of America

## Abstract

A plethora of magnetic nanoparticles has been developed and investigated under different alternating magnetic fields (AMF) for the hyperthermic treatment of malignant tissues. Yet, clinical applications of magnetic hyperthermia are sporadic, mostly due to the low energy conversion efficiency of the metallic nanoparticles and the high tissue concentrations required. Here, we study the hyperthermic performance of commercially available formulations of superparamagnetic iron oxide nanoparticles (SPIOs), with core diameter of 5, 7 and 14 nm, in terms of absolute temperature increase *ΔT* and specific absorption rate (*SAR*). These nanoparticles are operated under a broad range of AMF conditions, with frequency *f* varying between 0.2 and 30 MHz; field strength *H* ranging from 4 to 10 kA m^−1^; and concentration *c_MNP_* varying from 0.02 to 3.5 mg ml^−1^. At high frequency field (∼30 MHz), non specific heating dominates and *ΔT* correlates with the electrical conductivity of the medium. At low frequency field (<1 MHz), non specific heating is negligible and the relaxation of the SPIO within the AMF is the sole energy source. We show that the *ΔT* of the medium grows linearly with *c_MNP_*, whereas the *SAR_MNP_* of the magnetic nanoparticles is independent of *c_MNP_* and varies linearly with *f* and *H^2^*. Using a computational model for heat transport in a biological tissue, the minimum requirements for local hyperthermia (*T_tissue_* >42°C) and thermal ablation (*T_tissue_* >50°C) are derived in terms of *c_MNP_*, operating AMF conditions and blood perfusion. The resulting maps can be used to rationally design hyperthermic treatments and identifying the proper route of administration – systemic versus intratumor injection – depending on the magnetic and biodistribution properties of the nanoparticles.

## Introduction

The hyperthermic treatment of a malignant tissue is based on the deployment of sufficiently large heat doses over time to induce cell death or cell sensitization [1], [2]. Heat affects the architecture of the cell cytoskeleton; the molecular transport across the cell membrane and the function of receptors, in a dose and time dependent manner [3], [4], [5], [6], [7]. Two main treatments have been proposed: *hyperthermia* and *thermal ablation*. In regional hyperthermia, the malignant tissue is exposed to a temperature field slightly above 42°C for a relatively long time (a few hours) [8], [9], [10]. This is not sufficient *per se* to induce significant cell death and, therefore, is mostly used as an adjuvant treatment in support of conventional chemo- and radiation-therapy [11], [12], [13]. Differently, thermal ablation induces rapid cell death by exposing the malignant tissue to high temperature fields (>50°C) for a short time (a few minutes). Radiofrequency (RF) ablation is the most commonly used thermal ablation strategy [14], [15]. Although these approaches are clinically available and have shown some satisfactory results, they present important limitations due to their invasiveness and incomplete tumor destruction, allowing significant probability of cancer recurrence and metastasis [1], [16].

Nanoscale technologies offer multiple opportunities to improve the efficacy of hyperthermic treatments by enhancing specificity; reducing invasiveness; and providing multifunctional capabilities, such as imaging and drug delivery, synergistically. Gold-based (AuNPs); carbon-based (CNPs); and iron oxide nanoparticles (IONPs) are the most commonly used nanotechnological platforms for hyperthermic treatments. AuNPs can be tailored to absorb near-infra red (nIR) light and transform it into heat readily released in the surrounding tissue. This approach, known as *photothermal therapy*, has been applied to the treatment of different tumor types, leading to complete tumor regression in animal models [17], [18], [19], [20], [21]. Although AuNPs can generate locally high temperatures, well above 50°C [22], they suffer by two major limitations: the maximum penetration depth of nIR light in a biological tissue is of the order of a few millimeters, making this approach quite inefficient for the treatment of deep tumors; the lack of clinical imaging modalities for the in vivo detection of AuNPs. Carbon-based materials, such as fullerenes and carbon nanotubes, have shown very promising heating behaviors in nIR light and RF fields [23], [24]. However, their cytotoxicity is still highly debated and under careful scrutiny [25]. The third class of nanoparticles, IONPs, can be efficiently stimulated to generate heat by alternating magnetic fields (AMFs) [26]. With this approach there are no limitations in penetration depth, and clinical MRI has been routinely used to detect IONPs in humans [27], [28], [29]. Although several types of magnetic nanoparticles have been proposed, magnetite (Fe_3_O_4_) is by far the material that has been more extensively tested in clinical and clinically relevant settings demonstrating favorable biocompatibility and biodegradability [25], [30]. Protocols are available for large scale production of biocompatible magnetite nanoparticles and for their surface modification. For these reasons, Fe_3_O_4_ nanoparticles are the sole IONPs considered in this work.

For biomedical applications, sufficiently small nanoparticles are required generally exhibiting a total diameter not larger than 100 nm. Under this condition, the major mechanisms mediating the heat generation by IONPs exposed to AMFs are the Néel and Brownian relaxations, and hysteretic loses [26]. In nanoparticles with a magnetic core smaller than ∼20 nm, no more than one single magnetic domain is possible and relaxation becomes the sole dominating mechanism. This is the case of superparamagnetic iron oxide nanoparticles (SPIOs). Hysteretic loses dominate for nanoparticles with a larger magnetic core (20–100 nm) [31].

Most of the in vitro studies on the hyperthermic properties of IONPs have focused on maximizing the specific absorption rate (*SAR*), a parameter used to quantify the particle efficiency in converting electromagnetic energy into heat [28], [29], [32], [33], [34], [35], [36]. However, the *SAR* is not an intrinsic property of the nanoparticle in that, for a given IONP, it increases linearly with the frequency *f* and with the second power of the field strength *H* (*SAR* ∝*f × H^2^*) [36]. Consequently, very different *SAR* values have been published, ranging from 10^3^ to 10^6^ W kg^−1^, depending on the operating conditions (*H* = 1–100 kA m^−1^ and *f* = 100 kHz –50 MHz). This indeed generates confusion on the actual hyperthermic performance of magnetic nanoparticles. On the other hand, in vivo studies have mostly looked at tumor regression over time, upon single or multiple hyperthermic treatments. For instance, Shokier and coworkers [37] used 80 mg ml^−1^ of intratumorally injected ∼50 nm Fe_3_O_4_ nanoparticles. In another study, only 5 mg g^−1^ of tumor of ∼50 nm magnetite nanoparticles were exposed to high strength AMFs (*H = *55.7 kA m^−1^; *f = *150 kHz) for 10 min [38]. Kobayashi and collaborators incorporated 10 nm magnetite particles into cationic liposomes, treating the tumor tissue for 30 min at 46°C [39]. Notably, Jordan and collaborators have used 15 nm SPIOs for the ablation of Glioblastoma Multiforme upon intratumoral injection of 30 mg ml^−1^ of Fe and performing a 1 h treatment with AMFs at 10 kA m^−1^ and 100 kHz. The sole common factor among all these studies, and other here not cited, is the direct, intratumor injection of the magnetic nanoparticles.

In this work, three different commercial SPIO formulations are characterized for their hyperthermic performance under a wide range of *f-H* parameters, with *f* ranging from 100 kHz to 30 MHz and *H* varying from 4 to 10 kA m^−1^. Reproducing physiologically relevant conditions, the contribution of non specific heating over the specific, SPIO induced heating is systematically analyzed at high and low frequency fields. Then, computational modeling is used to predict the temperature field within a tumor as a function of the properties and concentration of the magnetic particles, and the local blood perfusion of the tissue. Heating of the surrounding healthy tissue is also identified as a function of time. Finally, the minimum requirements for cancer hyperthermic treatment are quantified in term of nanoparticle concentration and *SAR* values. Clinically relevant strategies for improving the delivery of nanoparticles within the tumor mass are also briefly discussed.

## Materials and Methods

### 1. Superparamagnetic Iron Oxide Nanoparticles (SPIOs) and their Characterization

Magnetite (Fe_3_O_4_) nanoparticles with a nominal magnetic core diameter of 5, 10, and 14 nm are purchased from Sigma-Aldrich (5 and 10 nm) and Genovis AB (14 nm). All nanoparticles are coated by a thin PEG (polyethylene glycol) layer. Before use, the samples provided by the manufactures were purified to remove aggregates following the steps described below. First, the samples were sonicated for 15 min and centrifuged for 6 minutes at 12,000 rpm; then the supernatant was collected, sonicated for 7 more minutes, and centrifuged again. Finally, the resulting supernatant was collected and used for the experiments. The Fe concentration of the final, purified colloidal suspension was measured using ICP-OES analysis (Inductively Coupled Plasma Optical Emission Spectrometer).

The magnetic core size is measured via Transmission Electron Microscopy (JEM-2100F TEM by JEOL Ltd.). Samples were diluted in DI water 10 times and 10 µL of the SPIO solution was deposited onto the surface of a TEM grid (Ted Pella, Inc., Form var/Carbon 400 mesh, Copper, approx. grid hole size: 42 µm) and left to dry for 1 h. The size distribution of the magnetic cores was estimated from TEM images considering at least 100 nanoparticles. The magnetic properties of the nanoparticles were investigated using a superconducting quantum interference device (SQUID) magnetometer (MPMS by Quantum Design Inc.). The saturation magnetization measurements were taken at 300 K with a field cycling from −5 to +5 T.

For ICP measurements, 150 µl sample solution was diluted in ∼1.5 ml of Nitric Acid (Sigma-Aldrich, 70%, purified by redistillation, ≥99.999% trace metals basis) and left to dry on a thermo plate at 110°C. This step was repeated twice. Finally the dried sample was diluted in 5 ml of a DI water solution at 2% Nitric Acid and filtered (0.22 µm pores size).

The electrical conductivity of solutions was measured using a z-potential (Malvern Instruments Ltd, Zetasizer Nano ZS). Briefly, a 20 µl sample solution was diluted in 750 µl of DI water and poured in disposable capillary cells provided by the same manufactures. Three repetitions of 16 runs and 3 minutes of delay between each repetition were performed.

### 2. Apparatus for Magnetic Hyperthermia

Two apparatus were used for the heating experiments under two different frequency ranges. High frequency field apparatus. This system was built to generate AMF fields at MHz frequency range (Radio Frequency) between 10 and 55 MHz, with amplitude up to 4 kA m^−1^. It consists of a LCR resonator as presented in the Figures S1a and S1c. The RF magnetic field in the solenoid of the resonator is measured using a small loop sensor. A frequency synthesizer along with an amplifier is used to drive input power into the resonator. The exciting coil and the resonator’s coil are critically coupled with 50 ohm impedance matching. At the resonant frequency, the AMF inside the coil is established. The main advantage of using such resonant circuit, besides the ability for generating different RF fields, is that the set up requires relatively low input power. This is due to high quality-factor Q of the LCR resonator, which provides enough additional RF field amplification. In this way RF energy is dissipated mainly in the resonator, not in the whole system, which simplifies possible problems with temperature stabilization and lowers significantly the requirements for the cooling system power. The capacitor in the LCR resonator is constructed with two 3 mm thick water cooled copper plates separated by single crystal sapphire (ε_r_ = 11) of thickness 12 mm. The two ends of the sapphire are cooled using plastic cuvettes allowing the flow of water. The resonator solenoid is made of six turns copper tube (3 mm outer diameter) wound into a coil. This has a diameter of 15 mm, a length of 21 mm and a distance per turn of 0.1 mm. Cooling water is pushed through copper tube. An additional piece of water-cooled single crystal sapphire (length 22 mm, width 12 mm, and height 45 mm) is housed inside the solenoid and works as a heat sink. In this design, a cylindrical quartz tube (inner diameter 2.5 mm, outer diameter 3 mm, height 20 mm) hosts 180 µl of sample and is mounted in a cylindrical hole drilled in a sapphire plate within the solenoid. Position of the sensor is controlled by micrometer positioner. Low frequency field apparatus. The apparatus produces AMFs in a discrete range of frequencies between 100 kHz and 1 MHz, with amplitude up to 10 kA m^−1^. The system is presented in the Figures S1b and S1d. The sample is inserted in a copper coil (inner diameter 50 mm), constructed from 4 mm copper tubing. The field coil is an element in a resonant RLC circuit with capacitance varying between 7 and 200 nF, and inductance varying in the range 4.5–9.1 µH, depending on the frequency. The coil quality factor Q is about 250. High quality RF capacitors and high purity copper coil are used in the system to minimize heat dissipation and enhance Q. The resonant frequency of the system can be changed by replacing the capacitor and/or coil. The field coil temperature is stabilized at 20±0.1°C by a thermoelectric water cooler/heater (ThermoCube 400, Solid State Cooling Systems Inc.). A cylindrical Plexiglas insert connected to a separate thermoelectric water cooler/heater (T251P-2, ThermoTec, Inc.) maintains an equilibrium temperature of sample holder at 19.8±0.1°C. A glass cylindrical tube (inner diameter 5 mm and length 35 mm) holds ∼700 µl of sample solution and is precisely mounted at the center of the field coil to minimize the effects of magnetic field inhomogeneities. For both apparatus, the temperature of the sample solution is measured every 1 s using a fiber optic GaAs temperature sensor (T1, Neoptix, Inc.) connected to a multichannel signal conditioner (Reflex, Neoptix, Inc.) with a resolution of 0.1°C. Note that metallic thermocouples must not be used in an inductive AMF in that they could lead to inaccurate temperature readings and SAR estimations.

### 3. Hyperthermia Experiments

The purified sample solutions (see above) were diluted in Milli-Q water to obtain concentrations of 0.1×, 0.3×, 0.5×, 0.7×, and 1× the original sample. The heating experiments were conducted under 4 different conditions: i) at ∼30 MHz and 4 kA m^−1^ for 400 s (high frequency field); ii) at ∼1 MHz and 5 kA m^−1^ for 20 min (low frequency field); iii) at ∼500 kHz and 10 kA m^−1^ for 20 min (low frequency field); iv) at ∼200 kHz and 9 kA m^−1^ for 20 min (low frequency field). Every sample solution was sonicated for 2 minutes before the actual experiment. The number of repetitions per group was six. The temperature sensor end was inserted vertically in the tube and was always cleaned with isopropanol before each single experiment. DI water was used for controlling the calibration of the apparatus periodically, at intervals of six experiments.

The outcome of each experiment is the temperature - time curve *T(t)* from which the maximum rise in temperature *ΔT* and the *SAR* of the solution can be readily extracted (see Text S1). The *SAR_f_* of the whole colloidal suspension (i.e. the fluid sample containing the magnetic nanoparticles) is given as.
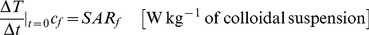
(1)where the first term on the left-hand side is the slope of the *T(t)* curve at t = 0 and *c_f_* is the heat capacity of the ferrofluid. Introducing the mass fraction 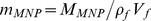, the *SAR_MNP_* of the sole magnetic nanoparticles is give as



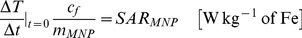
(2)The actual *SAR_f_* of the whole colloidal suspension is quantified using two approaches, namely the fitting and differential methods, as extensively explained in the Text S1.

### 4. Finite Element Modeling of the Temperature Field

The Pennes’ bioheat equation [40] was employed to quantify the temperature field within the domain of interest. The contribution of the magnetic nanoparticles was included as a distributed heat source. Similarly, blood perfusion of the tissue was modeled as a distributed heat sink within the computational domain. This is a square composed of two portions: a central region with the tumor tissue where magnetic nanoparticles could be laid uniformly (Ω_2_) and an outer region with the healthy tissue (Ω_1_). The whole region is surrounded by blood vessels, with which the healthy tissue in Ω_1_ exchanges heat [41]. The temperature evolution within the domain was described by the following set of equations.
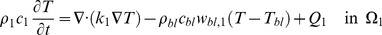
(3)

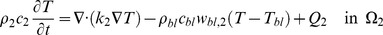
(4)

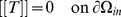
(5)

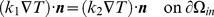
(6)

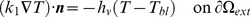
(7)

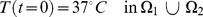
(8)where *Q_i_* (W mm^−3^) is the heat power density generated inside the region Ω_i_ *; k_i_, ρ_i_* and *c_i_* are respectively the thermal conductivity (W mm^−1^ K^−1^), the density (kg mm^−3^) and specific heat capacity (J kg^−1^ K^−1^) of the considered domain, Ω_i_, with *i = *1,2. All the quantities in Ω_2_ are defined according to the mixture theory, thus introducing the volume fraction 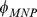, it follows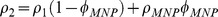, 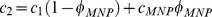 and 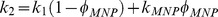. The perfusion parameters 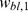 and 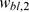 are the tissue perfusion rates (s^−1^) in Ω_1_ and Ω_2_ respectively, which have the following explicit expression, following a modified version of the model presented in [42], [43],



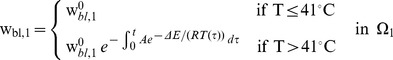
(9)

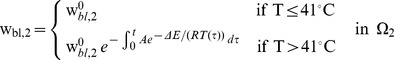
(10)Where *R* is the universal gas constant; *A* is the frequency factor; *ΔE* is the activation energy and 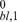 and 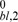 are the baseline perfusion values for the tissue (see Table S1).

Note that in Ω_1_ the sole source of heat was given by the non specific heating of the salts dispersed in the physiological fluids, whereas in Ω_2_ the specific contribution provided by the magnetic nanoparticles was also considered. Therefore, following the previous section 3 and Text S1, it follows
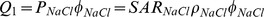
(11)

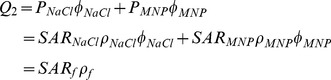
(12)where the pedex *f* indicates the ferrofluid. The system of equation (3) – (12) was solved using the finite element software (FEM) Comsol® (version 3.5a), with direct UMFPACK linear system solver. Relative and absolute tolerances used in calculations were 0.01 and 0.001, respectively. All computations were performed using a 2D square domain of 10 mm side, with 3816 triangular elements.

### 5. Statistical Analysis

Statistical analysis was performed using a Single Factor - ANOVA test with a significance level of 5%.

## Results

### 1. Physico-chemical Characterization of the SPIOs

**Figure 1:**
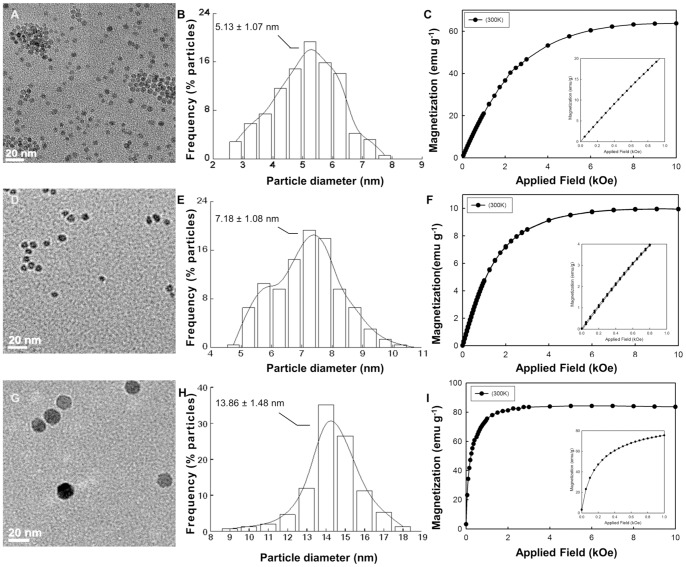
Physico-chemical characterization of the SPIO formulations. **(A, D, G)** TEM images of the three SPIO formulations. **(B, E, H)** Magnetic core size distribution as quantified from the TEM images. **(C, F, I)** Magnetic saturation of the SPIOs measured using a SQUID system (300 K). The insets in the figures show no appreciable hysteresis for all three formulations.

TEM micrographs of the three commercial SPIO preparations are presented in Figure 1a, 1d, and 1g. From the analysis of these images and considering over 100 nanoparticles, the size of the SPIO cores was measured to be 5.13±1.07 nm (nominal size: 5 nm); 7.18±1.08 nm (nominal size: 10 nm) and 13.86±1.48 nm (nominal size: 14 nm). The core size distribution is shown in the bar chart of Figure 1b, 1e, and 1h. Also, using a SQUID system, the magnetization curves were measured for all these nanoparticle formulations (Figure 1c, 1f, and 1i). Data showed no appreciable hysteresis (see insets) confirming the superparamagnetic behavior of the nanoparticles. Also, for the 5 and 14 nm SPIOs, large magnetic saturations were measured with *M_s_* ∼65 and 82 emu g^−1^, respectively; whilst far less performing were the 7 nm particles with an *M_s_* ∼10 emu g^−1^.

### 2. Hyperthermic Performance at High Frequency Field

**Figure 2:**
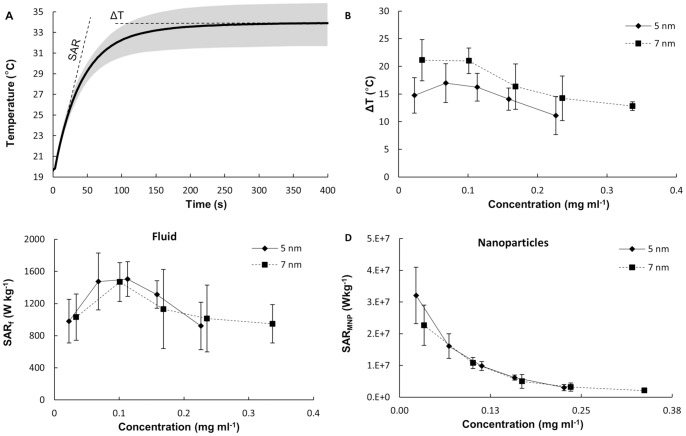
Hyperthermic performance at high frequency field (30 MHz). **(A)** A typical temperature-time curve from a hyperthermia experiment. The absolute temperature increase *ΔT* and *SAR* of the solution can be readily derived from this curve (data for 5 nm SPIOs; 0.02 mg ml^−1^ exposed to a 30 MHz and 4 kA m^−1^). **(B, C, D)** Absolute temperature increase *ΔT*; specific absorption rate of the solution (*SAR_f_*); and specific absorption rate of the magnetic nanoparticles (*SAR_MNP_*) as a function of the iron concentrations in solution, for the 5 and 7 nm SPIOs (30 MHz and 4 kA m^−1^).

Using the high-frequency field apparatus described in the Materials and Methods and Text S1, the hyperthermic properties of the SPIOs were characterized under different conditions at 30 MHz (RF regime). The temperature increase, *ΔT*, and the specific absorption rate, *SAR*, were extracted from the temperature (*T*) versus time (*t*) curves acquired continuously during the experiment. Results are presented in Figure 2, for the 5 and 7 nm SPIOs. A representative *T(t)* curve is given in Figure 2a, as derived for the 5 nm SPIOs exposed for almost 7 min to a field strength *H* = 4 kA m^−1^. *ΔT* is the increase in temperature from 20°C (ambient temperature) till equilibrium. Indeed, *SAR_f_* takes into account also the presence of the solution in which the particles are dispersed, whereas *SAR_MNP_* is introduced for characterizing the intrinsic hyperthermic properties of the SPIOs, as explained in the Materials and Methods and Text S1. The *ΔT*, *SAR_f_* and *SAR_MNP_* for the 5 and 7 nm SPIOs are plotted against the iron concentration in Figure 2b, 2c and 2d, respectively. A temperature increase of 15–20°C was observed over a wide range of nanoparticle concentrations, namely varying from 0.022 to 0.33 mg ml^−1^. The 7 nm particles heated up the solution slightly more than the 5 nm particles, but the difference was statistically not significant. Even more intriguing are the values derived for the *SAR_f_* and the *SAR_MNP_*. A biphasic behavior was observed for the *SAR_f_* with a maximum occurring at about 0.1 mg ml^−1^ (Figure 1c). On the other hand, the *SAR_MNP_* decreased continuously as the nanoparticle concentration increases from 0.022 to 0.33 mg ml^−1^. No statistically significant difference was observed between the 5 and 7 nm SPIOs, for both measured *SAR*. Based on the definitions of *SAR* and following the current literature [35], [36], [44], [45], [46], these results are intriguing in that it would have been expected i) a fixed *SAR_MNP_* independent of the iron concentration; ii) a steady growing *ΔT* and *SAR_f_* with the iron concentration; iii) statistically significance difference in *ΔT* and *SAR* between the 5 and 7 nm particles, which have very different magnetic properties (see Figure 1c and 1f).

### 3. Non Specific Heating at High Frequency Field

**Figure 3:**
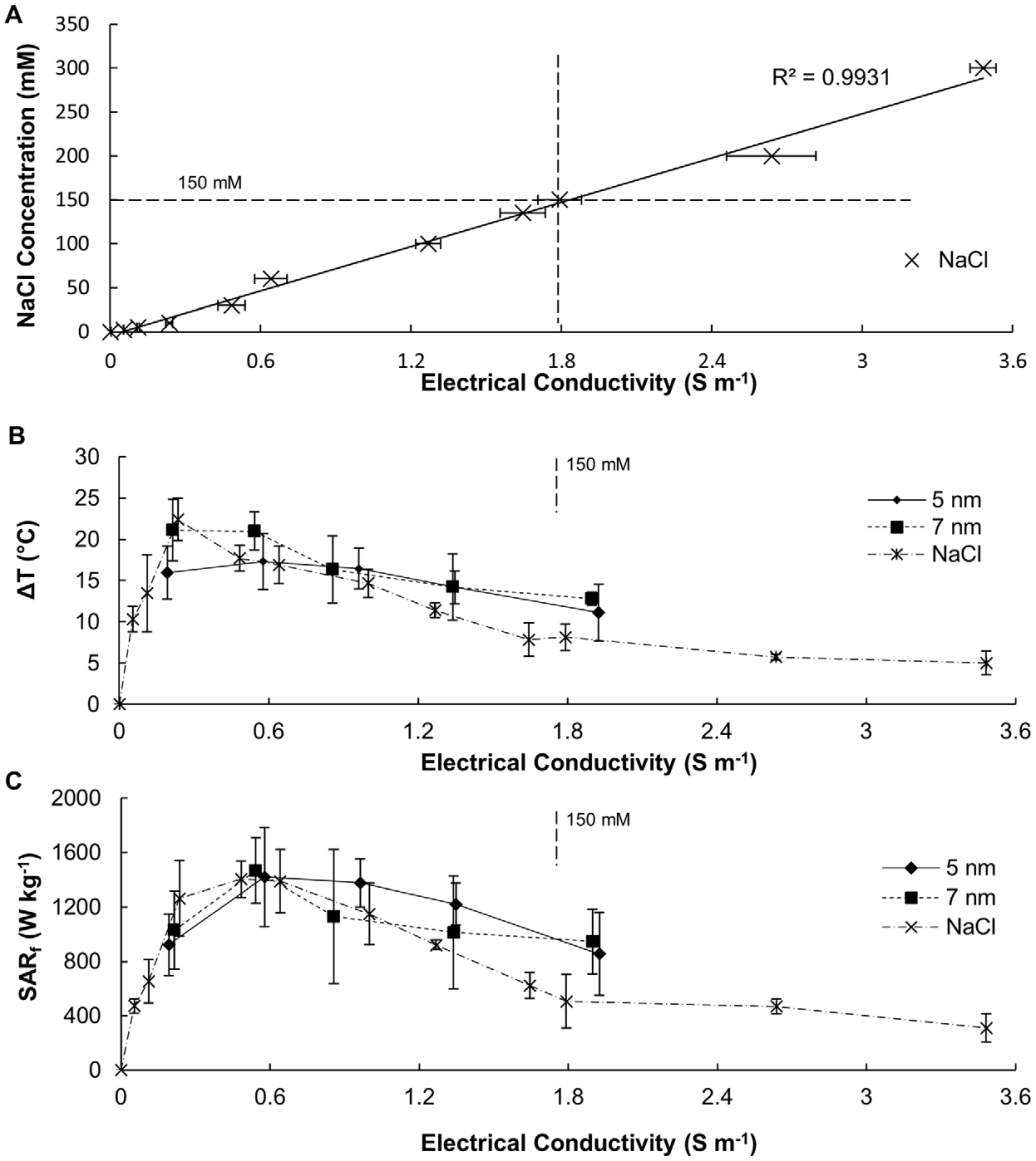
Non specific heating at high frequency field (30 MHz). **(A)** NaCl concentration against the electrical conductivity of the solution. **(B, C)** Absolute temperature increase *ΔT* and specific absorption rate (*SAR_f_*) as a function of the electrical conductivity for 5, 7 nm SPIOs and NaCl sample solutions (30 MHz and 4 kA m^−1^).

Puzzled by the results shown in Figure 2, the *ΔT* and *SAR_f_* values for a pure solution of NaCl were measured and compared to those registered for the SPIOs. This is shown in Figure 3. Different concentrations of NaCl were considered, namely ranging from 0 (pure, DI water) to 300 mM (supra-physiological salt concentrations). The electrical conductivity of the solutions was also measured by using a Z-potential instrument. As expected, Figure 3a shows a linear increase in the electrical conductivity of the NaCl solution with the salt concentration. Then, the *ΔT* and *SAR_f_* for the NaCl solutions at different salinities were measured using the same approach described above for the SPIOs. The results are presented in Figure 3b and 3c (dot-dashed line with cross) as a function of the electrical conductivity of the solution, rather than the salt concentration. Knowing that non-specific heating in the RF regime could be associated with ions dispersed in solutions [47], the electrical conductivity was also measured for the 5 and 7 nm SPIO solutions. Then, the data from Figure 2b and 2c were rephrased in terms of the electrical conductivity of the solution rather than its iron concentration. Thus, Figure 3b and 3c show the *ΔT* and *SAR_f_* variation over the electrical conductivity of the solutions with NaCl (dot-dashed line with cross), 5 nm SPIOs (solid line with diamond) and 7 nm SPIOs (dashed line with square).

The trends and absolute values for the three sets of data are very similar with almost no statistically significant difference within the range 0.05 to 1.89 S m^−1^. The intriguing biphasic behavior noted above for the SPIOs (Figure 2b–c) is here observed for the free NaCl solution too, implying that it might just be related to non specific rather than specific heating. This is in agreement with the behavior reported in [47], where solution enriched in NaCl are infused within the malignant tissue prior exposure to RF fields. Also, a statistically significant difference between the SPIOs and the NaCl solutions was only observed for larger electrical conductivities, namely >1.5 S m^−1^. Note that a physiological solution (150 mM of NaCl) exhibits an electrical conductivity of 1.8 S m^−1^. For ∼300 mM of NaCl, the electrical conductivity is 3.6 S m^−1^.

**Figure 4:**
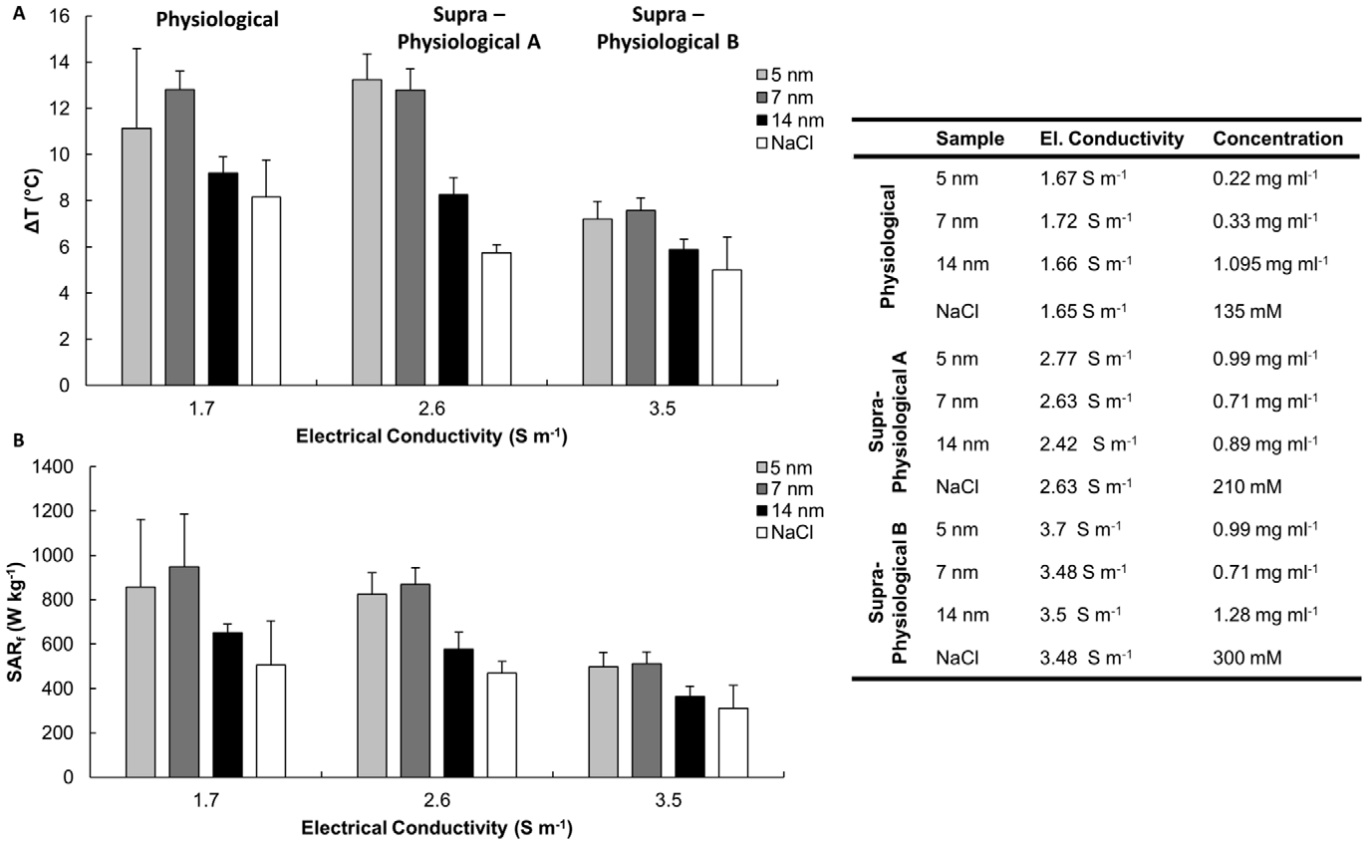
Mild heating of SPIOs at high frequency field (30 MHz). **(A, B)** A comparison between the absolute temperature increase *ΔT* and specific absorption rate (*SAR_f_*) of 5, 7, 14 nm SPIO and NaCl solutions at fixed electrical conductivities. The table provides the electrical conductivity and corresponding concentrations for the tested sample solutions (30 MHz and 4 kA m^−1^).

These differences are analyzed in more details in the bar charts of Figure S2 and Figure 4. The Supporting data confirm the non specific nature of the heating measured in Figure 3 by presenting the *ΔT* and *SAR* for diluted and centrifuged colloidal solutions.

The bar chart of Figure 4 shows the variation of *ΔT* and *SAR* for three different salt concentrations, namely 135 (physiological), 210 and 300 mM (supra-physiological), and the considered three SPIO preparations. A minimal difference between the 5 and 7 nm SPIOs and the NaCl solution was observed under these conditions, with a *SAR_f_* for the formers being two times larger than the latter (∼900 and 450 Wkg^−1^). No significant difference was observed even for the 14 nm SPIOs. These results confirmed that in the physiological and supra-physiological regime, the 5 and 7 nm SPIOs heat up the solution with *SAR* that are larger than the NaCl solution alone, but the contribution of non specific heating is comparable with the heat generated by the magnetic nanoparticles.

### 4. Hyperthermic Performance at Low Frequency Field

**Figure 5:**
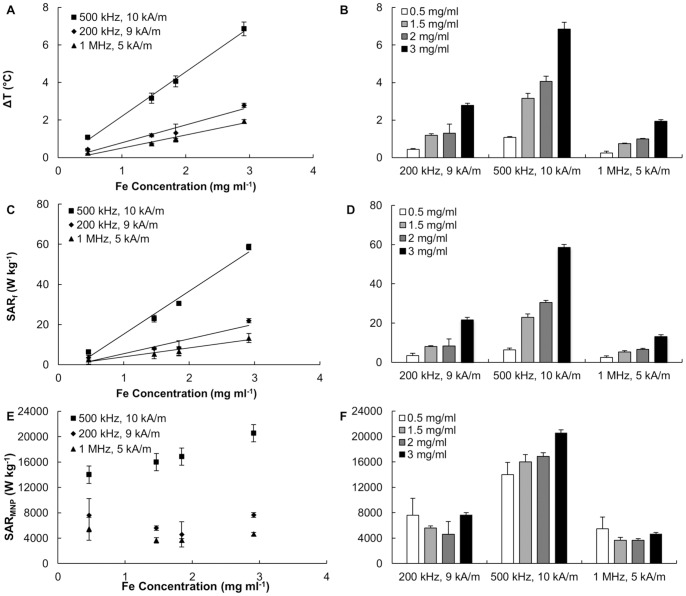
Hyperthermic performance at low frequency fields (200; 500; and 1,000 KHz). **(A, C, E)** Absolute temperature increase *ΔT*, specific absorption rate for the solution *SAR_f_*, and specific absorption rate of the magnetic nanoparticles *SAR_MNP_* as a function of the iron concentration under different AMF operating conditions (200 kHz and 9 kA m^−1^; 500 kHz and 10 kA m^−1^; and 1 MHz and 5 kA m^−1^). **(B, D, F)** Absolute temperature increase *ΔT* specific absorption rate for the solution *SAR_f_*, and specific absorption rate of the magnetic nanoparticles *SAR_MNP_* at three different AMF operating conditions for different SPIO concentrations. (0.5, 1.5, 2, and 3 mg ml^−1^).

The hyperthermic properties of the SPIOs were characterized at 200, 500 and 1,000 kHz using the second apparatus described in the Materials and Methods and Text S1 (Figures S1b–d). The data are plotted in Figure 5. No appreciable heat is generated by NaCl solutions, even at physiologically relevant salt concentrations, for all frequencies tested (Figure S3). This confirms that for sufficiently low frequencies, non specific heating is negligible. Next, the 5, 7 and 14 nm SPIOs were investigated under different conditions with a concentration of 0.4 mg ml^−1^. However, both the 7 and 14 nm SPIOs did not exhibit any significant heating at these lower frequencies, possibly due to the insufficient magnetization of the first particle and large size of the second. The low magnetic saturation value of 7 nm particles is an interesting observation and requires further experiments which are beyond the scope of this paper. The inferior performances of 14 nm particles can be attributed to their size and also to the surface coating of the particles [48].

The *ΔT* and *SAR_f_* of the 5 nm SPIOs showed a concentration and frequency dependent behavior, in agreement with the accepted theory [26]. Figure 5 shows a linear behavior of *ΔT* and *SAR_f_* with the Fe concentration, whereas the *SAR_MNP_* is almost constant over the considered range of concentrations. Also in Figure 5f, comparing the cases of *f = *200 and 500 kHz, it can be readily appreciated a *SAR_MNP_* increase of 2.5 times (from 6,400 to 16,800 W kg^−1^) consistent with the corresponding variation in *f* (*H* ∼10 kA m^−1^). Similar observations can be drawn for the other combinations of *f* and *H*.

From Figure 5, it results that 5 nm SPIOs at a concentration of 5 mg ml^−1^ generate a temperature increase *ΔT* of about 10°C after exposure to a 500 kHz field with a strength of just 10 kA m^−1^. It should be noted that field strengths up to 50 kA m^−1^ have been used in the literature [38].

These data demonstrate that at low frequencies (<1 MHz) non specific heating is virtually absent and significant increments in temperature can be generated and sustained for long periods of times. Note however that the maximum temperature increase depends on the volume of solution and the environmental conditions. Therefore, a feasibility analysis must also include the modeling of the heat generated from the metal nanoparticles, its transfer to the surrounding tissue, and corresponding temperature increase over time.

### 5. Computational Modeling of the Temperature Field within the Tissue

**Figure 6:**
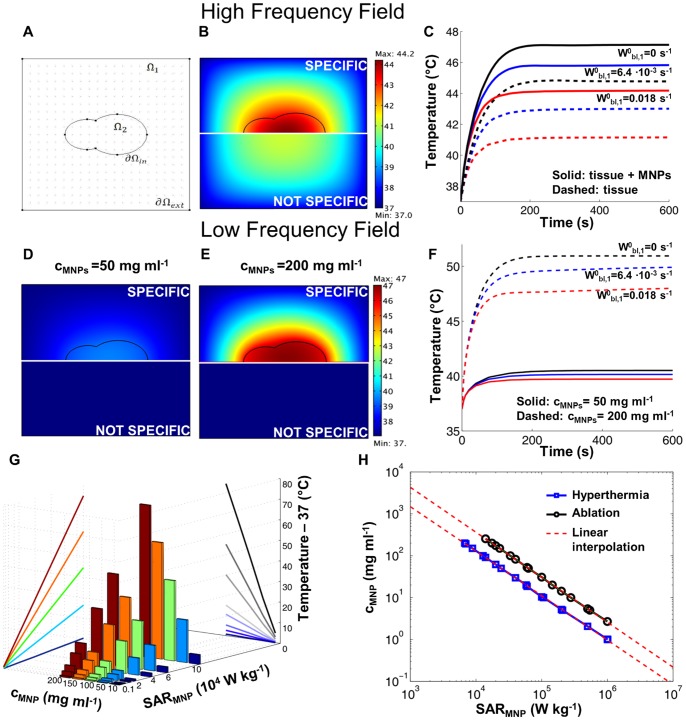
Computational modeling of the temperature field within a biological tissue. **(A)** Computational domain showing the tumor tissue (Ω_2_) surrounded by the healthy tissue (Ω_1_). **(B)** Temperature field at equilibrium for a uniform distribution of SPIOs in Ω _2_ (*SAR_f = _*900 W g^−1^) and non specific tissue heating in Ω_1
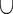
_Ω_2_ (*SAR_f_* = 450 W g^−1^) (blood perfusion 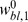 = 0.018 s^−1^). Bottom half-panels refer to non specific heating alone (*c_MNP_* = 0). **(C)** Temperature increase with time in the center of Ω_2_ for different blood perfusion levels in Ω_1_. Solid lines refers to specific heating (magnetic nanoparticles distributed in Ω_2_ and dashed lines refers to non specific heating alone. **(D, E)** Temperature field at equilibrium for a uniform distribution of SPIOs in Ω_2_ at two different concentrations (50 and 200 mg ml^−1^). Bottom half-panels refer to non specific heating alone (*c_MNP_* = 0). **(F)** Temperature increase with time in the center of Ω_2_ for different blood perfusion levels in Ω_1_. Solid lines and dashed lines refer to 50 and 200 mg ml^−1^ of SPIOs, respectively. **(G)** Maximum absolute temperaturereached in the tumor center for different nanoparticle concentrations, *c_MNP_* and specific absorption rates, *SAR_MNP_*. **(H)** Isotemperature linesfor tissue hyperthermia (*T_tissue_* = 42°C) and thermal ablation (*T_tissue_* = 50°C) drawn as a function of the nanoparticle concentration *c_MNP_* and specific absorption rate *SAR_MNP_*.

The temperature field is quantified using a Finite Element Method, as described in the Materials and Methods. A schematic representation of the computational domain is shown in Figure 6a. Here Ω_1_ is the healthy tissue surrounding the tumor located in Ω_2_, where the SPIOs are uniformly distributed. As per the performed experimental work, two conditions are modeled: high frequency with *f* = 30 MHz and low frequency with *f = *500 KHz. In the first case, a nanoparticle concentration of 0.22 mg ml^−1^, with *SAR_t_* = 450 W kg^−1^ and *SAR_f_* = 900 W kg^−1^ was considered as from the data reported in Figure 4b. The corresponding volumetric fraction 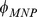 was about 4.3×10^−6^, thus it was reasonably assumed that the presence of the nanoparticles did not affect the physical properties of the tissue. The value for the heat exchange parameter 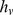 was derived referring to the heating of a pure NaCl solution (Figure 4a). Considering a single domain with 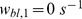 a heat exchange coefficient *h_v_* = 1.65×10^−3^ W mm^−2^ K^−1^ reproduces the observed 8°C increase in temperature for a NaCl solution with *SAR_NaCl_* = 450 W kg^−1^. Note that this value of 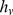 is in agreement with data presented in literature [49]. All the parameters used in the simulations are listed in Table S1.

The temperature field was derived by solving equation (3) and (4) for *T(* ***x*** *, t)*, considering different values of 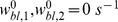, with boundary conditions (5)-(6)-(7) and initial condition (8). Figure 6b shows the temperature distribution within the computational domain at time t = 600 s, for 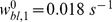. The upper portion of the panel gives the temperature field resulting from the heat generated by the SPIOs deposited in Ω_2_ in addition to the non-specific heating; whereas the bottom portion of the panel shows the results corresponding to the sole non-specific heating. There is clearly an increase in temperature within the tumor domain (Ω_2_) due to the presence of the SPIOs, however the temperature difference between the two conditions is only of about 3°C, being the max temperatures equal to ∼44 and ∼41°C, respectively (see Figure 6c, red lines). Also, the healthy tissue surrounding the tumor is exposed to significantly high heat doses with a minimum temperature of ∼42°C, deriving from the non-specific heating of the tissue as well as from the diffusion of heat from the tumor. Figure 6c shows the increase with time of the temperature in the center of the domain where the absolute maximum temperature is registered within the whole domain, for different perfusion levels of the healthy tissue. Note that within 200 s (∼3 min), the system reaches almost the steady state. In Figure 6c, the relevance of non-specific heating can be immediately deduced by comparing the data for the solid (specific heating) and dashed (non-specific heating) lines.

In the case of low frequency, the non-specific heating is negligible and heat is solely generated within the tumor domain Ω_2_ where the SPIOs are deposited. Therefore, *SAR_t_* = 0 W kg^−1^ everywhere within the computational domain. Considering the same geometry and parameters as for the high frequency case, the temperature field is provided in Figure 6d and 6e for two different SPIO concentrations, namely 50 and 200 mg ml^−1^. The *SAR_MNP_* used was extrapolated from experimental results presented in Figure 5e for the 5 nm SPIOs, assuming a linear increase of the specific absorption rate with the particle concentration. In both bottom panels of Figure 6d and 6e, no increase in temperature is observed (lack of non specific heating), whereas as expected in the upper panels the temperature increases with the concentration of the SPIOs. At the highest concentration of 200 mg ml^−1^, the maximum temperature of ∼47°C is reached quite uniformly within the tumor domain Ω_2_. In this case, the temperature increase within the healthy tissue has to be ascribed solely to heat transport from the central tumor domain Ω_2_. Figure 6f shows the increase with time of the temperature in the center of the domain for different perfusion levels. Even in this case, steady state conditions are reached within almost 200 s (∼3 min). Note that all these data correspond to the case in which no perfusion occurs within the tumor tissue, which indeed leads to slightly higher temperatures within the Ω_2_ domain.

Using the same computational framework, a systematic analysis can be performed to quantify the equilibrium temperature under different conditions, and in particular for different values of the SPIO concentration *c_MNP_* and *SAR_MNP_*. This is shown in the bar chart of Figure 6g, which confirms a steady, linear increase in the maximum temperature achieved in the tumor tissue with *c_MNP_* and *SAR_MNP_*. It is well accepted that tissue thermal ablation can be efficacious only by achieving temperatures equal or larger than 50°C for sufficiently long periods of time. Mild ablation or hyperthermia can be achieved at temperatures equal or larger than 42°C. Therefore, choosing these as target temperatures, two lines can be drawn in the *c_MNP_* - *SAR_MNP_* plane as shown in Figure 6h. These are quasi linear lines in a double logarithmic plot for the range of concentrations and specific absorption rates considered and are described by the equation.
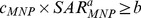
(13)where *a* = 1.0616 and *b* = 2.2714×10^6^ W m^−3^ for *T = *42°C; *a* = 1.0737 and *b* = 7.1565×10^6^ W m^−3^ for *T* = 50°C. Three characteristic operating regimes can be identified in Figure 6h: i) *insufficient heating* for *c_MNP_* - *SAR_MNP_* values falling below the hyperthermia curve (*T_eq_*<42°C); ii) *hyperthermia* for *c_MNP_* - *SAR_MNP_* values falling between the hyperthermia and ablation curves (42°C≤*T_eq_*<50°C); and iii) *ablation* for *c_MNP_* - *SAR_MNP_* values falling above the ablation curve (*T_eq_*≥ 50°C). Figure 6h provides a design map for rationally selecting the hyperthermic treatments and identifying the proper route of administration – systemic versus intratumor injection – depending on the magnetic and biodistribution properties of the nanoparticles. Note that this result is general in that it can be applied for any nanoparticle, including ferromagnetic particles, for which the SAR and local tissue concentration are known.

It should here be emphasized that nanoparticles, especially if systemically injected, would not distribute uniformly within the target tissue. Their concentration is expected to be larger at sites with higher vascular permeability and blood perfusion; and this will vary within the tumor mass as well as with the type and stage of the disease. Nonetheless, the assumption of a uniform distribution of nanoparticles within the target tissue provides a conservative estimation on the maximum temperature that can be reached within the region of interest, for a given total number of particles.

## Discussion

The data presented in Figure 6h demonstrate that to achieve tissue hyperthermia and thermal ablation, sufficiently large values of *SAR_MNP_* and SPIO concentrations are required. Therefore, it is here important to discuss strategies to improve the hyperthermic properties and tumor accumulation of magnetic nanoparticles.

**Figure 7:**
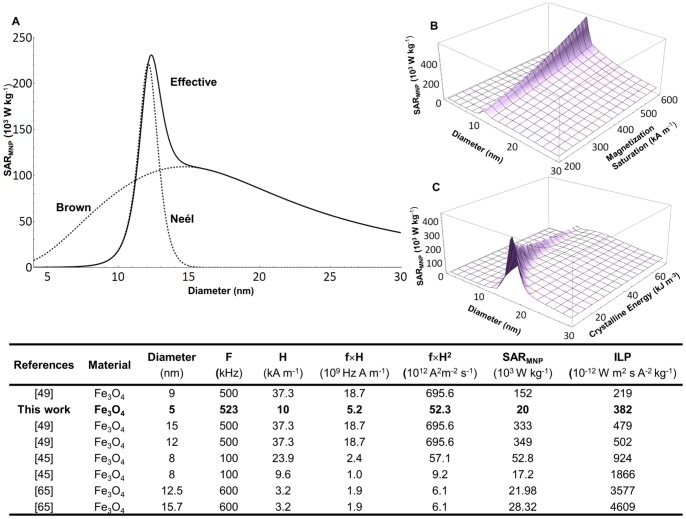
Modulating the *SAR_MNP_* of SPIOs. **(A)** Theoretical specific absorption rate of magnetic nanoparticles *SAR_MNP_* as a function of the diameter. The Brown and Neel relaxation curves are also presented. **(B, C)** The variation of *SAR_MNP_* with the magnetization saturation and crystalline energy of the SPIOs. The table lists *SAR* and *ILP* values for magnetite-based nanoparticles presented in the literature.

The *SAR_MNP_* depends on many factors including nanoparticle features, such as the size, shape and surface properties of the SPIOs, and the operating conditions of the AMF apparatus (frequency *f* and strength *H*). The effect of the particle size has been extensively studied and theories are available to predict the variation of *SAR_MNP_* with the diameter *d* of the nanoparticles. Following Rosensweig [26], an optimal particle size for each operating frequency *f* can be identified that would maximize *SAR_MNP_*, as shown in Figure 7a for *f* = 500 kHz and *H* = 10 kA m^−1^. The two contributions of the Brownian and Neel relaxation are also clearly presented. Note that the optimal nanoparticle size and its corresponding *SAR_MNP_* depend also on the intrinsic, material properties of the SPIO, such as the crystalline energy and magnetization saturation. This is addressed in the plots of Figure 7b and 7c, respectively, and it demonstrates that the maximum *SAR_MNP_* grows as the crystalline energy decreases and the magnetization saturation increases. Moreover, experiments have demonstrated that the heating efficiency of magnetic nanoparticles can be enhanced by proper design of their surfaces [48], [50], shape and composition selection [51], [52]. All this has led to the synthesis of nanoparticles with superior *SAR_MNP_* reaching values as high as 4×10^6 ^W kg^−1^ [50]. A list of the *SAR* values and other nanoparticle parameters is presented in the Table of Figure 7 for the SPIOs and in the Text S1 for several other formulations (Table S2).

Once the optimal nanoparticle properties have been established, the *SAR_MNP_* can be still tuned by controlling the operating conditions. Theoretical and experimental works have demonstrated that, within the AMF regime here of interest, the specific absorption rates would grow linearly with the frequency (∝*f*) and with the second power of the AMF strength (∝*H^2^*). This is also confirmed by the data presented in Figure 5. The list in the Table S2, shows values for the *SAR_MNP_* spanning over 5 orders of magnitude and ranging from 2×10^2^ to ∼4×10^6^ W kg^−1^ of Fe. Interestingly, this huge variation reduces significantly once the *SAR_MNP_* is normalized by the product *f*×*H^2^*, giving the intrinsic loss power (*ILP*) [35]. Limiting to the case of magnetite nanoparticles, the *ILP* ranges from ∼219×10^−12^ to 4,600×10^−12^ W m^2^ s A^−2^ kg^−1^ with the 5 nm SPIOs used in the present work providing a value of ∼382×10^−12^ W m^2^ s A^−2 ^kg^−1^. By increasing the field strength up to 50 kA m^−1^, the *SAR_MNP_* of the 5 nm SPIOs used here would grow up to ∼0.5×10^6^ W kg^−1^, and the corresponding concentrations needed for hyperthermia and ablation therapy would be ∼2 and 5.5 mg ml^−1^, respectively, as from equation (13). Based on this, the use of the parameter ILP seems more suitable to evaluate and compare the heating efficiency of metallic nanoparticles.

It is here important to recall the seminal work done by Atkinson and Brezovich on magnetic hyperthermia treatments and patient discomfort. In particular, Atkinson and colleagues [53] proposed a maximum limit for the product *f × H* = 4.85×10^8^ A turns m s^−1^. This number is based on discomfort measurements performed on patients over 20 years ago. In the same work, the authors have clearly reported that the maximum tolerable dose depends also on the equipment, duration, location and extension of the region of treatment; and on the specific patient. Therefore, the value 4.85×10^8^ A turns m s^−1^ should be considered as an indication rather than an absolute strict limit.

The majority of the in vivo experiments available in the literature deal with locally, intratumorally injected nanoparticles with concentrations ranging from a few mg ml^−1^ to a few hundreds of mg ml^−1^. Therefore, the questions should be posed on whether sufficient concentrations of SPIOs for hyperthermia and thermal ablation could be achieved within a tumor mass via systemic injection. A very elegant study on the biodistribution of radio-labeled SPIOs was published recently demonstrating tumor accumulation on the average of ∼1.0% ID g^−1^ for up to 70 nm in size nanoparticles [54]. This level of tumor accumulation for systemically injected nanoparticles could be enhanced by following two strategies: i) encapsulating SPIOs into larger carriers that are rationally designed to lodge within the diseased vasculature of tumors [55], [56] and ii) magnetically dragging the SPIOs within the tumor mass using external forces, generated by static magnetic fields [57], [58]. An issue that can arise when performing experiments on mice is non-specific major uptake of SPIOs in other organs, such as the liver or spleen. Accumulation here can highly exceed the level of 1% ID g^−1^ found in the tumor, causing unwanted damage if the whole mice body is exposed to AMFs. Nevertheless, in human application it is possible to localize the area of exposure with highly focused field, reducing the risk of unwanted damages. Cytotoxicity analysis conducted on SPIOs in mice, rats and humans have shown from mild to tolerable side effects up to concentrations of 4,000 mg Fe kg^−1^ [59], [60]. This would imply an injected dose of ∼100 mg Fe for a 20 g mouse. Thus, considering of 1% ID g^−1^ tissue accumulation data given above, a total SPIO concentration within the tumor mass of ∼1.0 mg Fe ml^−1^ would be expected (1 g ≈ 1 ml of tissue). For these levels of tumor accumulation, hyperthermia and thermal ablation could solely be achieved via the systemic injection of SPIOs with a *SAR_MNP_*∼10^6^–10^7^ W kg^−1^. This would be only one order of magnitude larger than the values obtained with commercially available 5 nm SPIOs stimulated at 500 kHz and 50 kA m^−1^. Referring to the Table S2, the nanoparticles presented in [50] with a *SAR_MNP_*∼4×10^6^ W kg^−1^ at 500 kHz and 37.3 kA m^−1^ could reach such high values by increasing the field strength to 50 kA m^−1^ (∼7.2×10^6^ W kg^−1^) and the minimum concentrations required for hyperthermia and ablation would be of 0.1 and 0.3 mg ml^−1^, respectively.

Based on the above reasoning, one would conclude that hyperthermia and thermal ablation of cancerous tissues are feasible with the systemic injection of magnetic nanoparticles for sufficiently high *SAR* values, as given by equation (13).

### Conclusions

Three commercially available formulations of superparamagnetic iron oxide nanoparticles (SPIOs) were characterized for their hyperthermic performance using Alternating Magnetic Fields (AMF) with a strength *H* ranging from 4 to 10 kA m^−1^ and frequency *f* varying from 0.2 to 30 MHz. The three formulations had different magnetic core diameters, namely 5, 7 and 14 nm, and the nanoparticle surface was coated with short PEG chains. The absolute temperature increase *ΔT* and specific absorption rate *SAR* were measured under different AMF operating conditions. In the high frequency regime (30 MHz), non specific heating, associated with the salts dispersed within the sample solution, dominated and a mild SPIO-induced heating was detected only at physiological and supra-physiological salt concentrations (≥ 150 mM). At lower frequencies (≤ 1 MHz), heating was solely generated by the relaxation of the SPIOs and no heating was measured for control, salt solutions. A mathematical model, based on the finite element discretization of the bioheat equation, was developed to predict the increase over time of the temperature field in a biological tissue. From this, two scaling laws in the form 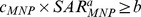 were derived to identify minimum requirements for local hyperthermia (*T_tissue_*>42°C; a = 1.0616, b = 2.2714×10^6 ^W m^−3^) and thermal tissue ablation (*T_tissue_*>50°C; a = 1.0737, b = 7.1565×10^6 ^W m^−3^). The resulting design maps can be used to rationally design hyperthermic treatments and select the proper route of administration – systemic versus intratumor injection – depending on the magnetic properties and biodistribution performance of the nanoparticles. The presented experimental results and in silico simulations would suggest that tumor tissue ablation is feasible also via the systemic administration of nanoparticles.

## Supporting Information

Figure S1 **Schematics of the circuit diagrams and images for the two apparatus.** **(A,** **C)** High frequency field system. **(B**, **D)** Low frequency field system. The table lists their operational conditions.
(TIF)

Figure S2 **Non Specific Heating at high frequency field.** Comparison of total temperature variation *ΔT* **(A)** and *SAR_f_* **(B)** for 5 and 7 nm SPIO formulation with NaCl solutions for: i) the original sample, as after purification; ii) supernatant after centrifugation; and iii) dilution in Milli-Q water. The table on the right reports Fe concentrations and electrical conductivities.
(TIF)

Figure S3 **Hyperhtermic performance at low frequency field.** Absolute *ΔT* **(A)** *SAR_f_* **(C)**, and *SAR_MNP_* **(D)** for control samples (DI water, 10 and 150 mM NaCl solutions) and colloidal suspension (∼2 mg ml^−*1*^) of 5, 7 and 14 nm particles measured at 500 kHz and 10 kA m^−1^.
(TIF)

Figure S4 **Magnetic field inhomogeneity and temperature field variation. (A)** Drops of highly concentrated SPIO solutions equally spaced on a petri dish, and **(B)** infraRed image of the temperature field during excitation with an AMF (500 kHz, 10 kA m^−1^). **(C)** Temperature variation over time for the 5 drops places on the petri dish. The inset provides a data over the first 40 s of heating. *SAR_f_* values and statistical analysis for the center spot, spot 4 and spot 3 are listed in the table.
(TIF)

Figure S5 **Quantification of** ***SAR_f_*** **from a temperature-time curve.** Two methods can be used to estimate the *SAR_f_*, namely the fitting and differential method. The left column presents data for a 7 nm SPIO solution at 0.23 mg ml^−1^ exposed to 30 MHz –4 kA m^−1 ^AMF; the right column presents data for a 5 nm SPIO solution at 3.5 mg ml^−1^ exposed to 0.5 MHz –10 kA m^−1^ AMF. **(A**, **C)** Experimental data and fitting curves for the sample temperature variation over time. **(B, D)** *SAR_f_* computed via the differential method as a function the time interval size *Δt*. The table provides a direct comparison between the two methods used for estimating the *SAR_f_*.
(TIF)

Table S1 **Parameters used in finite element simulations.**
(DOCX)

Table S2 **Properties of magnetic nanoparticles as presented in the literature.**
(DOCX)

Text S1 **Supporting Information.**
(DOCX)
