# Supplementary figures and images for: Design Maps for the Hyperthermic Treatment of Tumors with Superparamagnetic Nanoparticles
Source: PLoS One. 2013 Feb 25;8(2):e57332. doi: 10.1371/journal.pone.0057332 (PMC3581487; doi:10.1371/journal.pone.0057332)

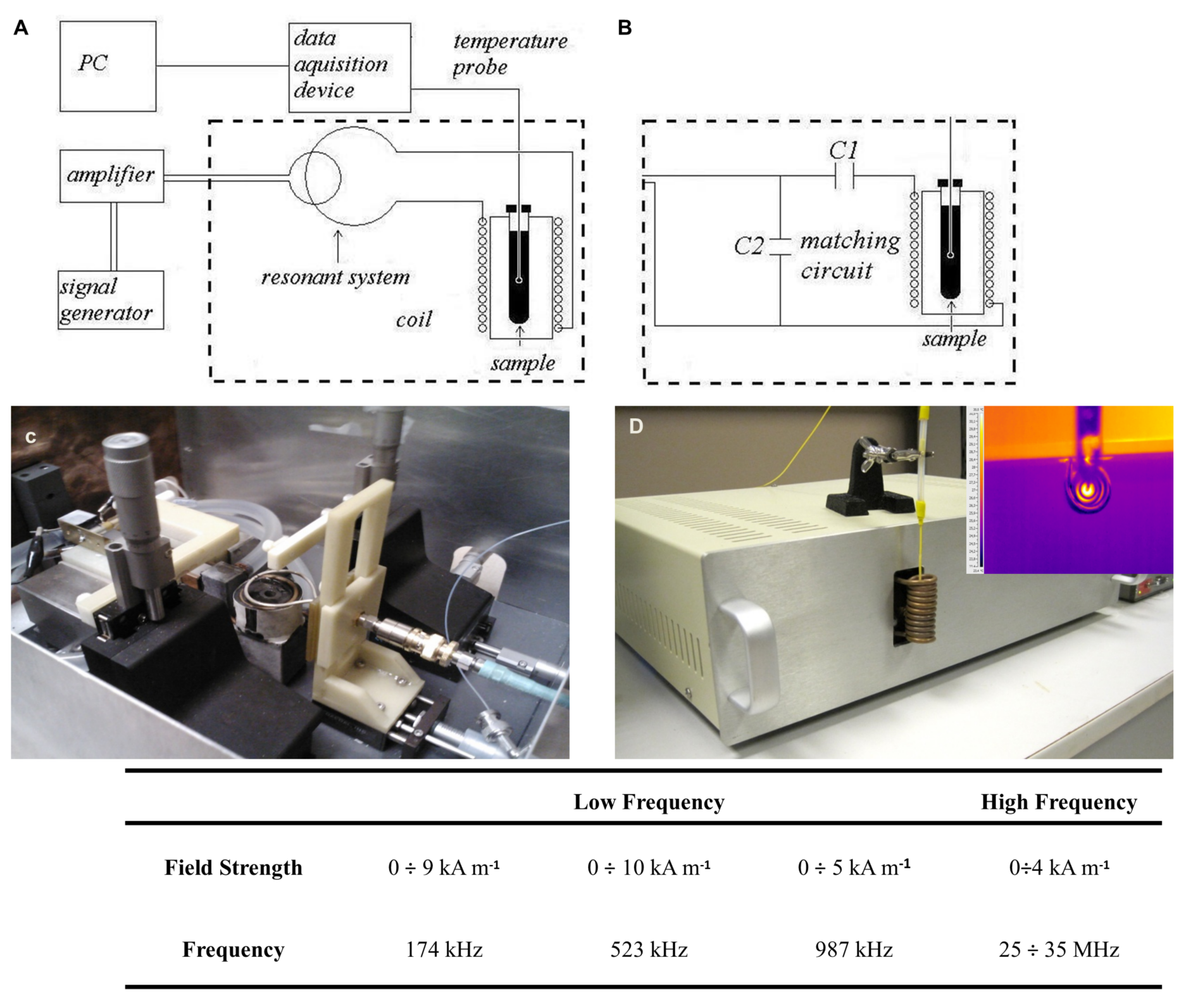

Supplement: Figure S1 — Schematics of the circuit diagrams and images for the two apparatus. (A, C) High frequency field system. (B, D) Low frequency field system. The table lists their operational conditions. (TIF) [file pone.0057332.s001.tif]

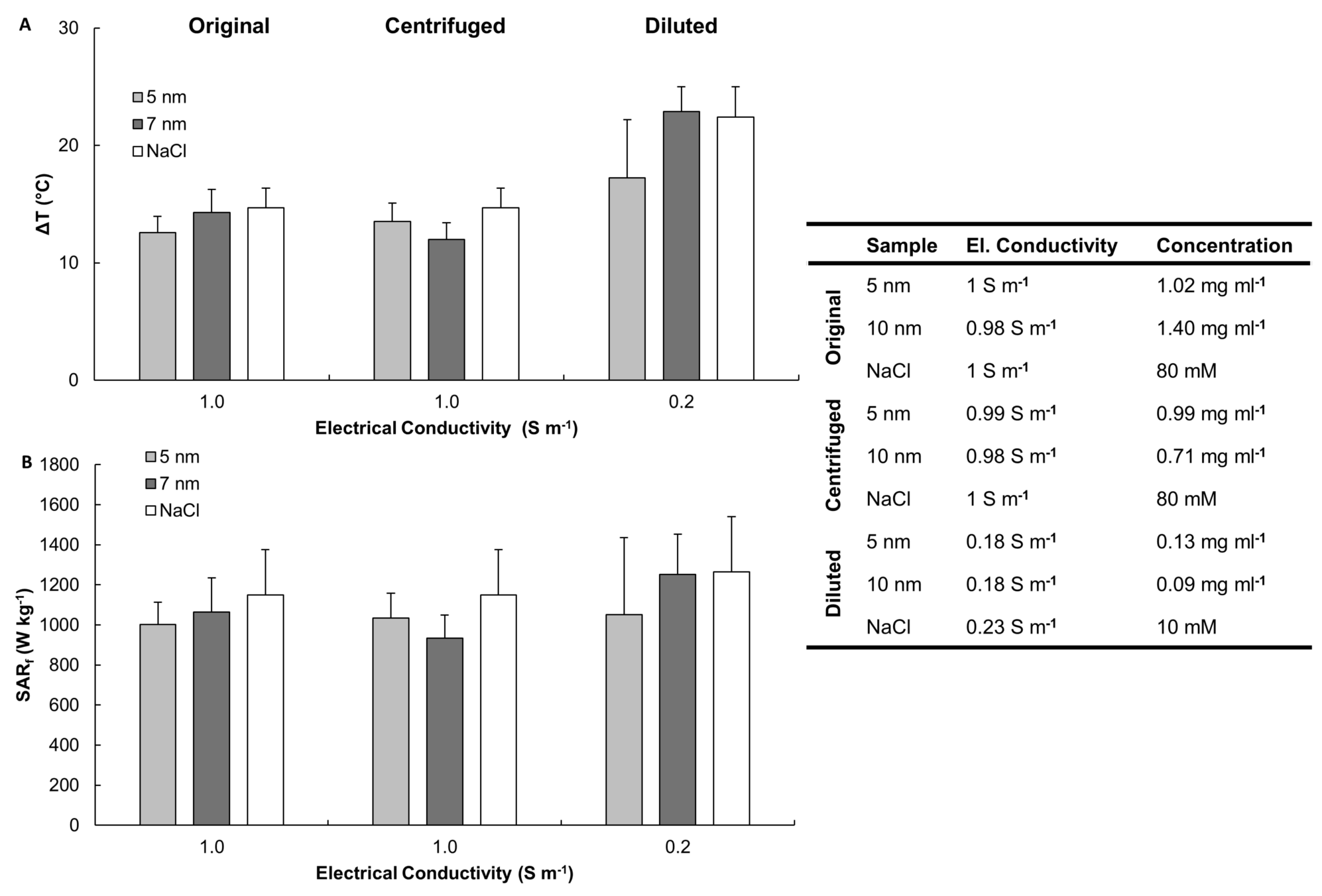

Supplement: Figure S2 — Non Specific Heating at high frequency field. Comparison of total temperature variation ΔT (A) and SARf (B) for 5 and 7 nm SPIO formulation with NaCl solutions for: i) the original sample, as after purification; ii) supernatant after centrifugation; and iii) dilution in Milli-Q water. The table on the right reports Fe concentrations and electrical conductivities. (TIF) [file pone.0057332.s002.tif]

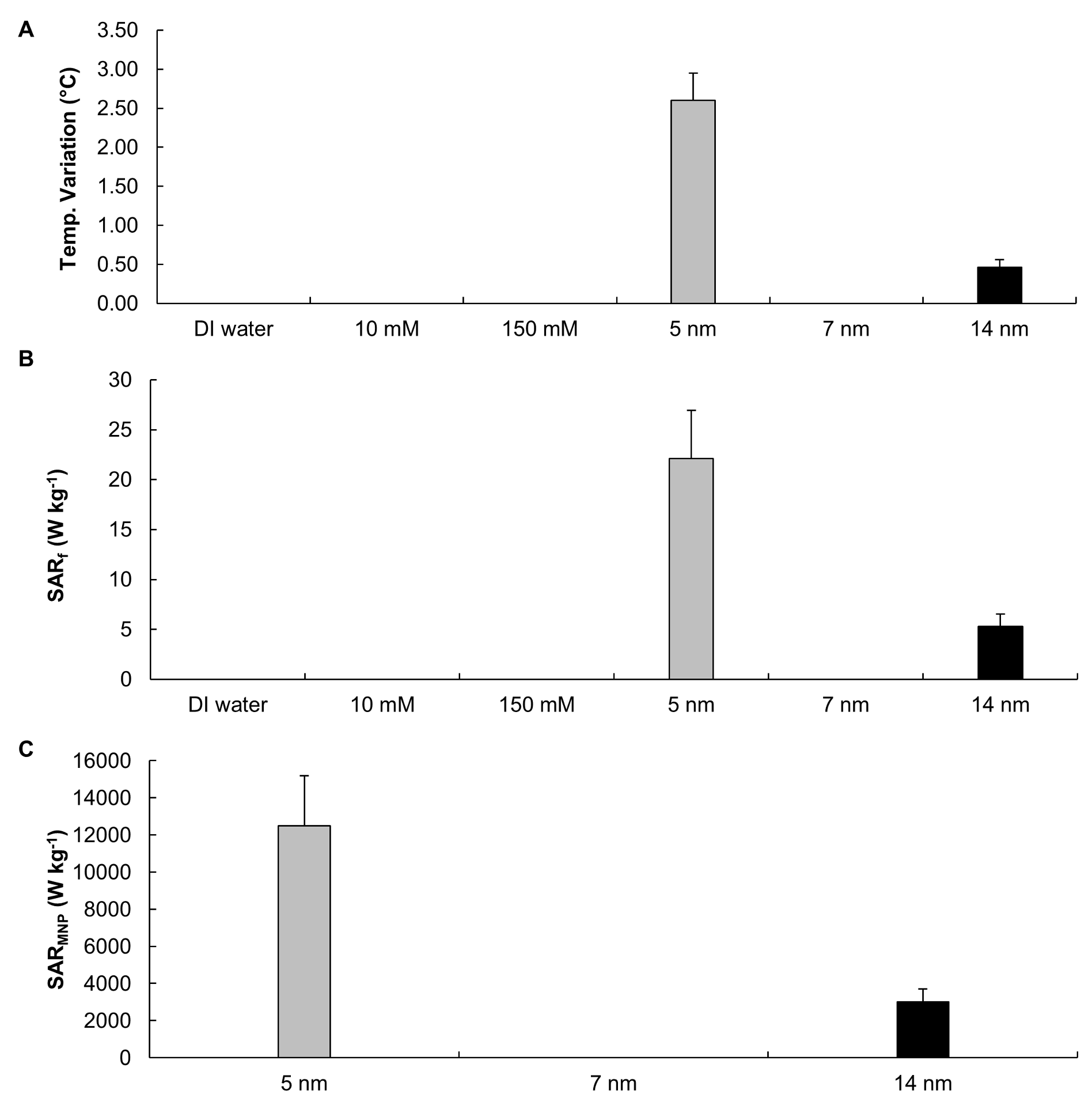

Supplement: Figure S3 — Hyperhtermic performance at low frequency field. Absolute ΔT (A) SARf (C), and SARMNP (D) for control samples (DI water, 10 and 150 mM NaCl solutions) and colloidal suspension (∼2 mg ml−1) of 5, 7 and 14 nm particles measured at 500 kHz and 10 kA m−1. (TIF) [file pone.0057332.s003.tif]

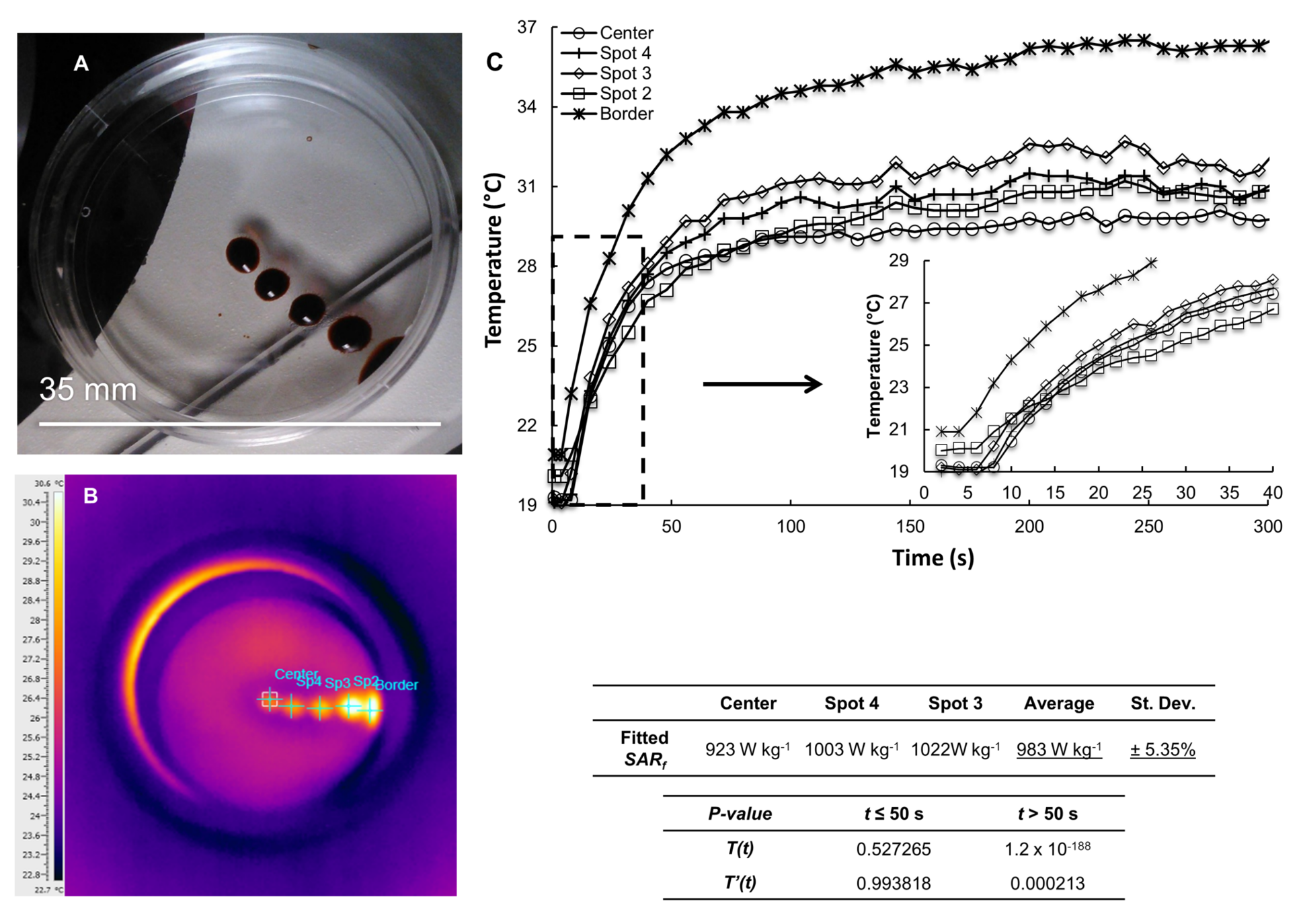

Supplement: Figure S4 — Magnetic field inhomogeneity and temperature field variation. (A) Drops of highly concentrated SPIO solutions equally spaced on a petri dish, and (B) infraRed image of the temperature field during excitation with an AMF (500 kHz, 10 kA m−1). (C) Temperature variation over time for the 5 drops places on the petri dish. The inset provides a data over the first 40 s of heating. SARf values and statistical analysis for the center spot, spot 4 and spot 3 are listed in the table. (TIF) [file pone.0057332.s004.tif]

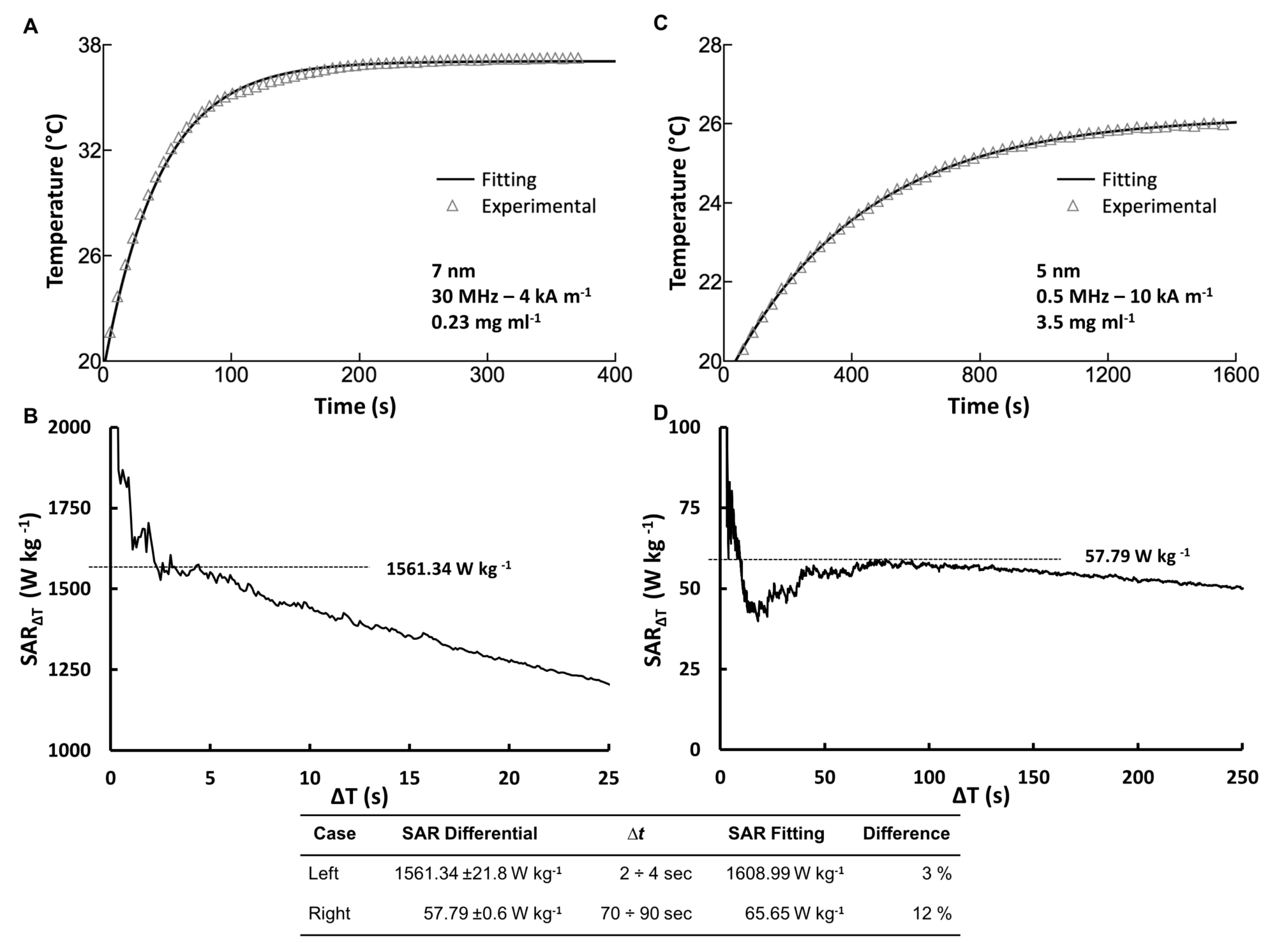

Supplement: Figure S5 — Quantification of SARf from a temperature-time curve. Two methods can be used to estimate the SARf, namely the fitting and differential method. The left column presents data for a 7 nm SPIO solution at 0.23 mg ml−1 exposed to 30 MHz –4 kA m−1 AMF; the right column presents data for a 5 nm SPIO solution at 3.5 mg ml−1 exposed to 0.5 MHz –10 kA m−1 AMF. (A, C) Experimental data and fitting curves for the sample temperature variation over time. (B, D) SARf computed via the differential method as a function the time interval size Δt. The table provides a direct comparison between the two methods used for estimating the SARf. (TIF) [file pone.0057332.s005.tif]
