# Supplementary material for: Design Maps for the Hyperthermic Treatment of Tumors with Superparamagnetic Nanoparticles
Source: PLoS One. 2013 Feb 25;8(2):e57332. doi: 10.1371/journal.pone.0057332 (PMC3581487; doi:10.1371/journal.pone.0057332)
Supplement: Table S1 — Parameters used in finite element simulations. (DOCX) [file pone.0057332.s006.docx]

**Table S1. Parameters used in finite element simulations.**

| Reference | Parameter | Parameter Description | Parameter Value |
| --- | --- | --- | --- |
| known | *T_bl_* | Blood temperature | 273.15+37 K |
| [[2](#_ENREF_2)] | *𝛒_bl_* | Blood density | 1060×10^-9^  kg mm^-3^ |
| [[3](#_ENREF_3)] | *𝛒_1_* | Tissue density | 1060×10^-9^  kg mm^-3^ |
| [[4](#_ENREF_4)] | *c_bl_* | Blood specific heat capacity | 3594 J kg^-1^ K^-1^ |
| [[5](#_ENREF_5)] | *c_1_* | Tissue specific heat capacity | 3470 J kg^-1^ K^-1^ |
| [[3](#_ENREF_3)] | *k_1_* | Tissue thermal conductivity | 0.51×10^-3^  W mm^-1^ K^-1^ |
| [[3](#_ENREF_3),[6](#_ENREF_6)] | *A* | Frequency factor | 1.98×10^6^ s^-1^ |
| [[3](#_ENREF_3),[6](#_ENREF_6)] | *𝚫E* | Activation energy | 6.67×105 J mol^-1^ |
| known | *R* | Universal gas constant | 8.314 J mol K^-1^ |
| From experiments | *SAR_1_* | Tissue Specific absorption rate | 450 W kg^-1^ |
| From experiments | *SAR_2_* | Tissue + MNPs Specific absorption rate | 900 W kg^-1^ |
| From experiments and in the range of [[7](#_ENREF_7)] | *h_v_* | Heat exchange coefficient | 1.65×10^-3^  W mm^-2^ K^-1^ |
| Derived by fitting calculations | *D* | Thermal resistance between particles and environment | Varying |
