# Supplementary material for: Design Maps for the Hyperthermic Treatment of Tumors with Superparamagnetic Nanoparticles
Source: PLoS One. 2013 Feb 25;8(2):e57332. doi: 10.1371/journal.pone.0057332 (PMC3581487; doi:10.1371/journal.pone.0057332)
Supplement: Table S2 — Properties of magnetic nanoparticles as presented in the literature. (DOCX) [file pone.0057332.s007.docx]

**Table S2. Properties of magnetic nanoparticles as presented in the literature.**

| **Reference** | | **Material** | **Diameter** | ***f*** | ***H*** | ***f*×*H*** | ***f*×*H^2^*** | ***SAR_MNP_*** | ***ILP*** |
| --- | --- | --- | --- | --- | --- | --- | --- | --- | --- |
|  | | | **[nm]** | **[kHz]** | **[kA m^-1^]** | **[10^9^Hz A m^-1^]** | **[10^12^ A^2^/m^-2^·s^-1^]** | **[10^3^W kg^-1^]** | **[10^-12^ W m^2^ s A^-2^kg^-1^]** |
| [[8](#_ENREF_8)] | γ-Fe_2_O_3_ | | 5.3 | 700 | 24.8 | 17.4 | 430.5 | 4 | 9 |
| [[8](#_ENREF_8)] | γ-Fe_2_O_3_ | | 6.7 | 700 | 24.8 | 17.4 | 430.5 | 14 | 33 |
| [[9](#_ENREF_9)] | γ-Fe_2_O_3_ | | 9 | 300 | 11.9 | 3.6 | 42.5 | 1.75 | 41 |
| [[9](#_ENREF_9)] | γ-Fe_2_O_3_ | | 25 | 300 | 11.9 | 3.6 | 42.5 | 2.5 | 59 |
| [[8](#_ENREF_8)] | γ-Fe_2_O_3_ | | 8 | 700 | 24.8 | 17.4 | 430.5 | 37 | 86 |
| [[10](#_ENREF_10)] | γ-Fe_2_O_3_ | | 10.9 | 70 | 3.4 | 0.2 | 0.8 | 0.2 | 247 |
| [[10](#_ENREF_10)] | γ-Fe_2_O_3_ | | 10.9 | 100 | 3.5 | 0.4 | 1.2 | 0.38 | 310 |
| [[10](#_ENREF_10)] | γ-Fe_2_O_3_ | | 10.9 | 185 | 2.3 | 0.4 | 1.0 | 0.52 | 531 |
| [[10](#_ENREF_10)] | γ-Fe_2_O_3_ | | 10.9 | 145 | 2.5 | 0.4 | 0.9 | 0.5 | 552 |
| [[8](#_ENREF_8)] | γ-Fe_2_O_3_ | | 10.2 | 700 | 24.8 | 17.4 | 430.5 | 275 | 639 |
| [[8](#_ENREF_8)] | γ-Fe_2_O_3_ | | 16.5 | 700 | 24.8 | 17.4 | 430.5 | 1650 | 3833 |
| [[11](#_ENREF_11)] | Zn0.4Co0.6Fe_2_O_4_@ Zn0.4Mn0.6Fe_2_O_4_ | | 15 | 500 | 37.3 | 18.7 | 695.6 | 3886 | 5586 |
| [[11](#_ENREF_11)] | MnFe_2_O_4_@ CoFe_2_O_4_ | | 15 | 500 | 37.3 | 18.7 | 695.6 | 3034 | 4361 |
| [[11](#_ENREF_11)] | MnFe_2_O_4_ | | 15 | 500 | 37.3 | 18.7 | 695.6 | 441 | 634 |
| [[12](#_ENREF_12)] | Fe_3_O_4_:Fe_2_O_3_ in 3:2 ratio | | 13 | 215 | 3.8 | 0.8 | 3.1 | 28 | 9019 |
| [[11](#_ENREF_11)] | Fe_3_O_4_ | | 9 | 500 | 37.3 | 18.7 | 695.6 | 152 | 219 |
| **This work** | **Fe_3_O_4_** | | **5** | **523** | **10** | **5.2** | **52.3** | **20** | **382** |
| [[11](#_ENREF_11)] | Fe_3_O_4_ | | 15 | 500 | 37.3 | 18.7 | 695.6 | 333 | 479 |
| [[11](#_ENREF_11)] | Fe_3_O_4_ | | 12 | 500 | 37.3 | 18.7 | 695.6 | 349 | 502 |
| [[13](#_ENREF_13)] | Fe_3_O_4_ | | 8 | 100 | 23.9 | 2.4 | 57.1 | 52.8 | 924 |
| [[13](#_ENREF_13)] | Fe_3_O_4_ | | 8 | 100 | 9.6 | 1.0 | 9.2 | 17.2 | 1866 |
| [[14](#_ENREF_14)] | Fe_3_O_4_ | | 12.5 | 600 | 3.2 | 1.9 | 6.1 | 21.98 | 3577 |
| [[14](#_ENREF_14)] | Fe_3_O_4_ | | 15.7 | 600 | 3.2 | 1.9 | 6.1 | 28.32 | 4609 |
| [[11](#_ENREF_11)] | Fe_2_O_4_@ CoFe_2_O_4_ | | 15 | 500 | 37.3 | 18.7 | 695.6 | 2795 | 4018 |
| [[11](#_ENREF_11)] | CoFe_2_O_4_@ MnFe_2_O_4_ | | 15 | 500 | 37.3 | 18.7 | 695.6 | 2280 | 3278 |
| [[11](#_ENREF_11)] | CoFe_2_O_4_@ Fe_2_O_4_ | | 15 | 500 | 37.3 | 18.7 | 695.6 | 1120 | 1610 |
| [[8](#_ENREF_8)] | Co-Fe_2_O_4_ | | 3.9 | 700 | 24.8 | 17.4 | 430.5 | 40 | 93 |
| [[11](#_ENREF_11)] | Co-Fe_2_O_4_ | | 9 | 500 | 37.3 | 18.7 | 695.6 | 100 | 144 |
| [[8](#_ENREF_8)] | Co-Fe_2_O_4_ | | 9.1 | 700 | 24.8 | 17.4 | 430.5 | 360 | 836 |
| [[15](#_ENREF_15)] | Bionized nanoferrite | | 28 | 150 | 85.9 | 12.9 | 1106.8 | 209 | 189 |
| [[15](#_ENREF_15)] | Bionized nanoferrite | | 28 | 150 | 85.9 | 12.9 | 1106.8 | 537 | 485 |
