# Supplementary material for: Design Maps for the Hyperthermic Treatment of Tumors with Superparamagnetic Nanoparticles
Source: PLoS One. 2013 Feb 25;8(2):e57332. doi: 10.1371/journal.pone.0057332 (PMC3581487; doi:10.1371/journal.pone.0057332)
Supplement: Text S1 — Supporting Information. (DOCX) [file pone.0057332.s008.docx]

**Supporting Information**

**Design maps for the hyperthermic treatment of tumors with superparamagnetic nanoparticles**

Antonio Cervadoro, Chiara Giverso, Rohit Pande, Subhasis Surangi,

Luigi Preziosi, Jarek Wosik, Audrius Brazdeikis, Paolo Decuzzi*

** corresponding author, E-mail: pdecuzzi@tmhs.org*

**Hyperthermia experiments and the definition of *SAR*.** The outcome of a magnetic hyperthermia experiment is the temperature versus time curve *T(t)* from which the maximum rise in temperature *ΔT* and the Specific Absorption Rate (*SAR_f_*) of the solution can be readily extracted. The first parameter, *ΔT*, quantifies the heat provided to the solution but it does depend on the specific experimental conditions, such as the geometry and material properties of the vial containing the solution, the vial insulation and ambient temperature. Differently, the *SAR_MNP_* is commonly used in literature to quantify the efficiency of metallic nanoparticles in transforming electromagnetic energy into heating.

In a typical hyperthermia experiment, the temperature of the solution grows steadily with time till new equilibrium is reached between heat deposition from the metallic nanoparticles and heat dissipation throughout the surrounding environment. Therefore, imposing energy conservation it results

| $PV_{MNP}-q=\frac{dT}{dt}c_{f}\rho_{f}V_{f}$ | [W] | (S1) |
| --- | --- | --- |

where *P* is the power loss density associated with the nanoparticles [1]; *V* is the volume; *q* is the heat exchanged with the environment; *T* is the sample temperature at time *t*; *c* and *ρ* are the heat capacity and density of the solution, respectively. The pedex *f* refers to ferrofluid, whereas *MNP* is used for the magnetic nanoparticles.
Note that the bio-heat equation described in the Materials and Methods of the main manuscript presents a more general mathematical description of the above problem. Introducing the volume fraction of the ferrofluid $\varphi=V_{MNP}/V_{f}$, after substituting back into equation (S1), it follows

| $\frac{P\varphi}{\rho_{f}}-\frac{q}{\rho_{f}V_{f}}=\frac{dT}{dt}c_{f}$ | [W kg^-1^] | (S2) |
| --- | --- | --- |

where *q* depends on the temperature difference between the ferrofluid (sample) and the surrounding environment. Therefore in the limit of *t* = 0, *q* is negligibly small and equation (S2) takes the form

| $\frac{P\varphi}{\rho_{f}}=\left.\frac{\Delta T}{\Delta t}\right\vert_{t=0}c_{f}=SAR_{f}$ | [W kg^-1^ of colloidal suspension] | (S3) |
| --- | --- | --- |

which represents the definition for the *SAR* of the whole ferrofluid (see equation (1) in the main manuscript). Dividing both terms of equation (S2) by the mass fraction$m_{MNP}=M_{MNP}/\rho_{f}V_{f}$, and passing to the limit for *t=0*, it follows

| $\frac{P}{\rho_{MNP}}=\left.\frac{\Delta T}{\Delta t}\right\vert_{t=0}\frac{c_{f}}{m_{MNP}}=SAR_{MNP}$ | [W kg^-1^ of Fe] | (S4) |
| --- | --- | --- |

which represents the definition for the *SAR* of the magnetic nanoparticles alone (see equation (2) in the main manuscript). Note that *SAR_MNP_* does not depend on the concentration of the nanoparticles but solely on *P* and the nanoparticle density. Also, equation (S3) and (S4) show that the corresponding *SAR* can be readily estimated by measuring the slope of the *T(t)* curve at t = 0.

The two *SAR* definitions are related through the equation

| $SAR_{f}\frac{V_{f}}{M_{MNP}}\rho_{f}=\left.\frac{\Delta T}{\Delta t}\right\vert_{t=0}c_{f}\frac{V_{f}\rho_{f}}{M_{MNP}}=\left.\frac{\Delta T}{\Delta t}\right\vert_{t=0}c_{f}\frac{1}{m_{MNP}}=SAR_{MNP}$ | (S5) |
| --- | --- |

**The apparatus for magnetic hyperthermia.** Two apparatus were used for the heating experiments under two different frequency regimes. The high frequency field apparatus is shown in the Figures.S1a and S1c; and the low frequency field apparatus is shown in the Figures.S1b and S1d. A constant temperature water chiller (Fisher Scientific) is used to render a constant sink temperature of 19.8 °C. Temperature was measured by a sensor inserted in the middle of the tube and is recorded versus time.

**Non specific heating at high frequency field.** In Figure.S2, the behavior of two particle formulations (5 and 7 nm SPIOs) are considered and compared with NaCl solutions. In particular, the original samples, provided by the vendor in a concentration of about 1.0 mg ml*^-1^* and with an electrical conductivity of 1 S m*^-1^*, were centrifuged and the supernatant was collected and exposed to an AMF (centrifuged samples). Although, the concentration of iron in the centrifuged solution is lower than in the original sample (see table in Figure.S2), no statistically significant difference is observed for *ΔT* and *SAR_f_*. Indeed, centrifugation process does not alter the ionic concentration so that the electrical conductivities of the original and centrifuged samples are the same. Moreover, the original samples were diluted in DI water to about five times their volume, thus reducing the iron, the ionic concentration and consequently the electrical conductivity to about 0.2 S m*^-1^* (diluted sample). In this case, a slight variation in *ΔT* and *SAR_f_* is observed as compared to the original sample, which is in line with the change in electrical conductivity (see Figure.3 in the main manuscript).

**Non specific heating at low frequency field.** Using the low frequency field apparatus, the hyperthermic properties of the SPIOs were characterized at 200; 500; and 1,000 kHz. No appreciable heat is generated by NaCl solutions, even at physiologically relevant salt concentrations, for all frequencies tested, as show in Figure.S3. This confirms that for sufficiently low frequencies non specific heating is negligible.

**Magnetic field inhomogeneity.** In order to assess variations in temperature due to the possible inhomegeneity of the magnetic field, a series of experiments were performed to measure the temperature variation associated with 5 drops (~6 μl) of highly concentrated SPIO solution, deposited in 5 different spots within the region of interest (magnetic coil). The drops were equally spaced and deposited radially on a petri dish, placed within the spires of the solenoid (magnetic coil). The drops had a diameter of ~ 3 mm, with a separation distance of ~1 mm (Figures.S4a and S4b). During the heating experiment, temperature maps were acquired using an IR camera (Figure.S4b) and the temperature *T(t)* was monitored continuously at the 5 different spots (Figure.S4c). It appears from the *T(t)* curves that the temperature for the spot at the border of the petri dish, which is more spread and insulated by the plastic wall of the petri dish, grows more rapidly in time and reaches higher values as compared with the four other spots. Conversely, the temperature variation associated with the center spot and the adjacent spot 4 and spot 3 are very similar. This can also be easily appreciated by observing the inset of Figure.S4c, where the temperature field is provided over the first 40 s of operation. Testing for statistical significance, it results that there is no statistically significant difference (*p* >> 0.05) between the temperature at the center spot and the temperature at spots 4 and 3, for *t* ≤ 50 s. However, as time increases, statistically significant differences between the three temperature fields appear, mostly because of the different heat exchange conditions. The same analysis has been perfomed on the temperature first derivative with time, which is directly proportional to the *SAR*, and identical conclusions can be drawn. Finally, the actual *SAR_f_* for all thee central spots is estimated providing the values 923, 1003 and 1022 W kg^-1^, moving from the center to spot 3. The percentage variation between these three values is within about 5%.

Note that the spot 3 is ~10 mm away from the center of the dish, whereas for the hyperthermia experiments in our manuscript, the sample solution was in a vial with an inner diameter of 5 mm (see the “Experimental Section”), firmly placed in the center of the solenoid. This would suggest that within the region of interest magnetic field inhomogeneity, and corresponding temperature variation, could be neglected.

**Computing the Specific Absorption Rate.**  Two methods were employed to quantify the SAR of the sample solution: i) *fitting method*, where the temperature variation *T(t)* with time is fitted over the whole duration of the experiment and then used to estimate the derivative *T’(0)* at time *t* = 0 s; ii) *differential method*, where a few experimental points next to the origin (*t* = 0 s) are considered to estimate the derivative *T’(0)*, exploiting the very mathematical definition of first order derivative and differential quotient

| $T'(t)=\frac{dT(t)}{dt}=\lim_{\Delta t\to0} \frac{T(t+\Delta t)-T(t)}{\Delta t}$ | (S6) |
| --- | --- |

For the fitting method, the first order ordinary differential equation (S1) for the heat transport is solved with respect to the temperature *T(t)* to give

| $T_{f}(t)=T_{a}+\frac{B}{A}(e^{At}-1)$ | (S7) |
| --- | --- |

where *T_a_* is the temperature at time *t* = 0 s and the fitting parameters *A* and *B* are

$$A=\frac{-1}{c_{f}\rho_{f}DV_{f}};\begin{matrix} & \end{matrix} B=P\varphi/c_{f}\rho_{f}$$

where *D* is the thermal resistance between the inner fluid and the outer environment. Note that the term *A* is related to the heat exchange with the surrounding environment; whereas the term *B* defines the heating generated by the nanoparticles in the solution.
For the differential method, the variation of the temperature increment (and related *SAR*) was estimated as a function of the time interval *Δt* based on the formula

| $SAR_{\Delta t}=c_{f}\frac{T(0+\Delta t)-T(0)}{\Delta t}$ | (S8) |
| --- | --- |

where *c_f_* is the heat capacity of the sample solution (*c_f_* = 4150.3 J kg^-1^ K^-1^).

Two different representative cases are here presented where sample solutions with different particle sizes (5 and 7 nm), concentrations (low and high) and field properties (low and high frequencies and field strengths) were exposed to AMF for different time periods (Figure.S5). The triangles identify the experimental points whereas the solid lines provide the interpolating curve derived by fitting the experimental data with the equation (S7) above.

For the *fitting method*, the parameters *A* and *B* and the corresponding statistical analysis were provided by using Mathematica as summarized in the table below:

| 5 nm 3.5 mg/ml 500 kHz | | | | |
| --- | --- | --- | --- | --- |
| Parameter | Value | Standard Error | t-Statistic | P-Value |
|  |  |  |  |  |
| A | 0.002356 | 2.07 ×10^-6^ | 1136.25 | 4.4 ×10^-2428^ |
| B | 0.015818 | 1.05 ×10^-5^ | 1499.92 | 1.3×10^-2630^ |

| 7 nm 0.23 mg/ml 30 MHz | | | | |
| --- | --- | --- | --- | --- |
| Parameter | Value | Standard Error | t-Statistic | P-Value |
|  |  |  |  |  |
| A | 0.022644 | 7.31 ×10^-5^ | 309.629 | 6.8×10^-451^ |
| B | 0.387681 | 1.13 ×10^-3^ | 341.854 | 7.8×10^-467^ |

The good agreement between the experimental and fitting parameter is demonstrated by the low p-values. By computing the first derivative with time at *t* = 0 s for the interpolating *T(t)* curve, the *fitting method* returns a *SAR* of 1608.99 and 65.65 W kg^-1^ for the two considered configurations.

For the *differential method*, the Figures.S5b and S5d show the variation of the *SAR_Δt_* as a function of the time interval *∆t* based on the equation (S8). Note that as the *∆t* reduces, the corresponding *SAR_Δt_* progressively increases almost linearly with *∆t* till it reaches a time interval in which is relatively constant and independent of *∆t*. For smaller time intervals, *SAR_Δt_* starts to change erratically and tends to increase rapidly as *∆t* approaches zero. For even smaller *∆t*, the temperature increase with respect to the ambient value *T(0)* is small and environmental factors induce quite significant fluctuations. This explains the erratic variation observed as *∆t* tends to zero. On the other hand, for large time intervals *∆t*, the corresponding *SAR_Δt_* decreases steadily in that the equilibrium temperature *T(0+∆t)* is approached with no more significant variation in the increment *T(0+∆t) – T(0).* The plots in the Figures.S5b and S5d clearly demonstrate that there is an intermediate range of *∆t* providing a constant *SAR_Δt_*, quite independent of *∆t*: this corresponds to the actual *SAR*. Note also that the proper time interval *∆t* over which *SAR_Δt_* is constant depends on the experimental conditions. Following the differential method, the *SAR* is estimated to be 1561.34 ± 21.8 and 57.79 ± 0.6 W kg^-1^ for the two considered configurations.

The percentage difference for the *SAR* values computed via the differential and interpolating methods is generally within about 10%, as detailed in the table in Figure.S5.


**References**

1. Rosensweig RE (2002) Heating magnetic fluid with alternating magnetic field. J Magnetism Mag Mats 252: 370-374.

2. J. D. Cutnell, Johnson. KW (1997) Physics, 4th Edition; 1, editor: John Wiley & Sons Canada, Ltd.

3. Gasselhuber A, Dreher MR, Negussie A, Wood BJ, Rattay F, et al. (2010) Mathematical spatio-temporal model of drug delivery from low temperature sensitive liposomes during radiofrequency tumour ablation. Int J Hyperthermia 26: 499-513.

4. Blake AS, Petley GW, Deakin CD (2000) Effects of changes in packed cell volume on the specific heat capacity of blood: implications for studies measuring heat exchange in extracorporeal circuits. Br J Anaesth 84: 28-32.

5. Faber P, Garby L (1995) Fat content affects heat capacity: a study in mice. Acta Physiol Scand 153: 185-187.

6. Brown SL, Hunt JW, Hill RP (1992) Differential thermal sensitivity of tumour and normal tissue microvascular response during hyperthermia. Int J Hyperthermia 8: 501-514.

7. dos Santos I, Haemmerich D, Pinheiro C, da Rocha A (2008) Effect of variable heat transfer coefficient on tissue temperature next to a large vessel during radiofrequency tumor ablation. BioMedical Engineering OnLine 7: 21.

8. Jean-Paul Fortin CW, Jacques Servais, Christine Ménager, Jean Claude Bacri, and Florence Gazeau (2007) Size-Sorted Anionic Iron Oxide Nanomagnets as Colloidal Mediators for Magnetic Hyperthermia. J AM CHEM SOC 129.

9. Baker I, Zeng Q, Li W, Sullivan CR (2006) Heat deposition in iron oxide and iron nanoparticles for localized hyperthermia. Journal of Applied Physics 99: 08H106.

10. Beković M, Hamler A (2010) Determination of the Heating Effect of Magnetic Fluid in Alternating Magnetic Field. IEEE TRANSACTIONS ON MAGNETICS 46: 4.

11. Lee J-H, Jang J-t, Choi J-s, Moon SH, Noh S-h, et al. (2011) Exchange-coupled magnetic nanoparticles for efficient heat induction. Nature Nanotechnology 6: 418-422.

12. Chen S, Chiang C-l, Hsieh S (2010) Simulating physiological conditions to evaluate nanoparticles for magnetic fluid hyperthermia (MFH) therapy applications. Journal of Magnetism and Magnetic Materials 322: 247-252.

13. Li Z, Kawashita M, Araki N, Mitsumori M, Hiraoka M, et al. (2010) Magnetite nanoparticles with high heating efficiencies for application in the hyperthermia of cancer. Materials Science and Engineering: C 30: 990-996.

14. Suto M, Hirota Y, Mamiya H, Fujita A, Kasuya R, et al. (2009) Heat dissipation mechanism of magnetite nanoparticles in magnetic fluid hyperthermia. Journal of Magnetism and Magnetic Materials 321: 1493-1496.

15. Dennis CL, Jackson AJ, Borchers JA, Ivkov R, Foreman AR, et al. (2008) The influence of magnetic and physiological behaviour on the effectiveness of iron oxide nanoparticles for hyperthermia. Journal of Physics D: Applied Physics 41: 134020.
